# Seasonal variation in SARS-CoV-2 transmission in temperate climates: A Bayesian modelling study in 143 European regions

**DOI:** 10.1371/journal.pcbi.1010435

**Published:** 2022-08-26

**Authors:** Tomáš Gavenčiak, Joshua Teperowski Monrad, Gavin Leech, Mrinank Sharma, Sören Mindermann, Samir Bhatt, Jan Brauner, Jan Kulveit

**Affiliations:** 1 Centre for Theoretical Studies, Charles University, Prague, Czech Republic; 2 Future of Humanity Institute, University of Oxford, Oxford, United Kingdom; 3 Faculty of Public Health and Policy, London School of Hygiene and Tropical Medicine, London, United Kingdom; 4 Department of Health Policy, London School of Economics and Political Science, London, United Kingdom; 5 Department of Computer Science, University of Bristol, Bristol, United Kingdom; 6 Department of Statistics, University of Oxford, Oxford, United Kingdom; 7 Department of Engineering Science, University of Oxford, Oxford, United Kingdom; 8 Oxford Applied and Theoretical Machine Learning (OATML) Group, Department of Computer Science, University of Oxford, Oxford, United Kingdom; 9 Faculty of Medicine, School of Public Health, Imperial College London, London, United Kingdom; 10 Department of Public Health, University of Copenhagen, Copenhagen, Denmark; Fundação Getúlio Vargas: Fundacao Getulio Vargas, BRAZIL

## Abstract

Although seasonal variation has a known influence on the transmission of several respiratory viral infections, its role in SARS-CoV-2 transmission remains unclear. While there is a sizable and growing literature on environmental drivers of COVID-19 transmission, recent reviews have highlighted conflicting and inconclusive findings. This indeterminacy partly owes to the fact that seasonal variation relates to viral transmission by a complicated web of causal pathways, including many interacting biological and behavioural factors. Since analyses of specific factors cannot determine the aggregate strength of seasonal forcing, we sidestep the challenge of disentangling various possible causal paths in favor of a holistic approach. We model seasonality as a sinusoidal variation in transmission and infer a single Bayesian estimate of the overall seasonal effect. By extending two state-of-the-art models of non-pharmaceutical intervention (NPI) effects and their datasets covering 143 regions in temperate Europe, we are able to adjust our estimates for the role of both NPIs and mobility patterns in reducing transmission. We find strong seasonal patterns, consistent with a reduction in the time-varying reproduction number *R*(*t*) (the expected number of new infections generated by an infectious individual at time *t*) of 42.1% (95% CI: 24.7%—53.4%) from the peak of winter to the peak of summer. These results imply that the seasonality of SARS-CoV-2 transmission is comparable in magnitude to the most effective individual NPIs but less than the combined effect of multiple interventions.

## Author Summary

Building on two state-of-the-art observational models and datasets, we adapt a fully Bayesian method for estimating the association between seasonality and SARS-CoV-2 transmission in 143 temperate European regions. This approach overcomes limitations of previous analyses that do not account for the implementation of non-pharmaceutical interventions (NPIs) or mobility during the first year of the pandemic and hence may yield biased estimates of seasonal effects. We find that the seasonality of SARS-CoV-2 transmission is comparable in magnitude to the most effective individual NPIs but less than the combined effect of multiple interventions. Our findings provide valuable insights for long-term modelling and policy planning. As seasons change, it is vital that policymakers employ accurate estimates of seasonal effects. In the summer, reductions in transmission that owe to seasonality should not be misattributed to population immunity. In the winter, policymakers must avoid anticipating a greater reduction due to seasonality than will actually occur.

## 1 Introduction

Since the onset of the COVID-19 pandemic, the role of seasonal variation in SARS-CoV-2 transmission has received significant scientific and political attention [1]. Understanding seasonal patterns is vital, as it enables more accurate inferences about current trends in transmission and how they may change over the longer term. For example, a proper understanding of seasonality can help policymakers avoid attributing declining incidence over the summer to population immunity alone, when in fact seasonality may be playing a meaningful role.

While seasonal variation is well-established for many respiratory viral infections [2], and some studies have suggested associations between temperature, humidity, and COVID-19 incidence [3–7], other analyses have failed to show a robust role of climate and weather [8, 9], particularly when population immunity is low [10]. A recent review found that the evidence remains inconclusive [11].

A further complication is that temperature, humidity, and UV radiation plausibly affect transmission and incidence through a range of biological and epidemiological mechanisms [2, 12]. These include virus stability and viability [13, 14], host susceptibility and immune response [15, 16], human behaviour [17, 18], and social factors such as holidays and school calendars [19, 20]. This multitude of plausible causal pathways makes it exceedingly difficult to disentangle the influence of various seasonal factors, particularly given the extensive multi-collinearities and interactions between environmental, biological, and behavioural elements [21, 22]. S1 Fig shows an overview of the various causal pathways, including existing literature and evidence on the collinearities between various factors. As Lofgren *et al.* note in the context of influenza, “*the myriad theories accounting for seasonality (…) suggest that the elegant and predictable periodicity of nonpandemic influenza is caused by a less-than-straightforward interaction of many different factors*,” meaning that “*recognition of this complexity, as well as the likelihood that seasonality arises from many different factors, is essential for continued examination and elucidation of seasonality*” [17].

Given the severe methodological challenge of disentangling these interrelated factors, a more tractable solution is to approach seasonality holistically with the purpose of understanding its overall effects. In this study, we infer a single seasonality parameter, describing the amplitude of the yearly variation in the time-varying reproduction number, *R*(*t*), for one climate region. While this single parameter does not disentangle the individual effects that comprise a seasonal profile, it accounts for the overall magnitude of the seasonal effect on SARS-CoV-2 transmission and thereby provides valuable insights for long-term policy planning.

Since both COVID-19 incidence and the presence of governmental non-pharmaceutical interventions have waxed and waned in consecutive waves since early 2020, adjusting for NPI effects is crucial for any effort to infer the influence of seasonality on transmission, yet early analyses of environmental drivers have largely not done so [5, 7, 11].

Some studies of environmental factors have taken indirect approaches to avoiding the influence of NPIs on their environmental estimates. In a recent study of the association between humidity, temperature, and SARS-CoV-2 transmission in Europe and North America, Landier *et al*. exclude from their analysis any periods at least 28 days after the implementation of ‘lockdown measures’ [4]. Ma *et al.* do include periods where measures are in place, however, instead of directly utilising data on NPIs, they use smoothed spatial and temporal splines to indirectly adjust for their influence [3]. Smith *et al.* compare the role of temperature in the presence and absence of ‘lockdown’ in the United States but only include a binary measure of whether stay-at-home orders were in place [22].

By contrast, we directly adjust for the influence of specific interventions by extending two hierarchical Bayesian models of NPI effects from Brauner *et al.* [23] and Sharma *et al.* [24] to include a term representing the multiplicative seasonal influence on the effective reproduction number.

Employing a common technique in infectious disease modelling [25, 26], we assume the seasonal variation itself is described by a sinusoidal modulation. We re-analyse data from the aforementioned two studies [23, 24] while restricting the scope to European regions in the temperate climate zone, where we assume the seasonality effect to be comparable both in its environmental and behavioural causal components.

## 2 Methods

We build on two previously published, state-of-the-art NPI effectiveness models: Brauner *et al.* [23] and *Sharma et al.* [24]. To estimate the seasonal variation in transmission while adjusting for the effect of NPIs, we extend these “base models” to include a seasonality effect. We fit each of the two resulting “seasonal models” separately, on separate data (see below); we thus obtain two distinct estimates for the seasonality effect.

### 2.1 Models

#### 2.1.1 Non-pharmaceutical intervention effectiveness models

In this section, we give a short summary of the common core of both base models. Please refer to Brauner *et al.* [23] and *Sharma et al.* [24] for more detailed descriptions of the models and their differences. Additionally, see S2 Appendix for a schematic diagram of the model structures.

Both models use a data-driven, cross-region modelling approach, in which a Bayesian hierarchical model is fitted jointly to a large set of regions. The models estimate NPI effects by comparing the timing of interventions in each region to the subsequent numbers of cases or deaths. Because each region deployed various combinations of interventions in different orders and with different outcomes, this method can disentangle the effect of individual interventions.

More concretely, the models use case and death data from each region to “backward” infer the number of new infections at each point in time, which is then itself used to infer the reproduction numbers. The NPI effects are then estimated by relating the daily reproduction numbers to the active NPIs, across all days and regions. This relatively simple, data-driven approach makes it possible to sidestep the assumptions about contact patterns and intensity, infectiousness of different age groups, and so forth, which are typically required in modelling studies.

**Fig 1:**
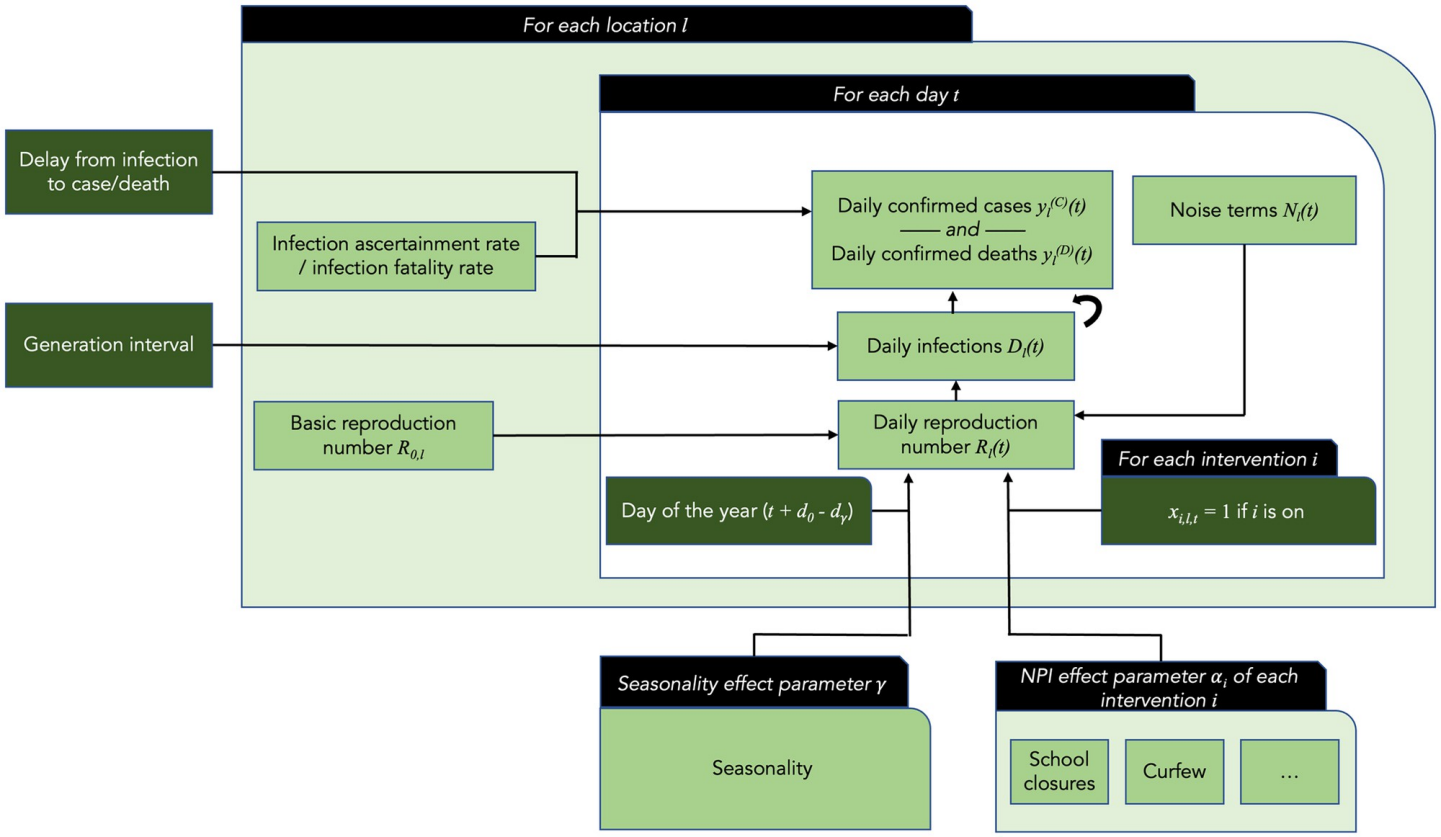
High-level overview of the common structure of both models used in this study. Dark nodes are observed, light nodes are inferred. See S2 Appendix for a detailed model graph for each individual model

Fig 1 shows the basic model structure, including our adaptation to account for seasonality.

Both models assume the time-varying reproduction number *R*(*t*) to be a product of *R*_0_, the “natural” reproduction number when no NPIs are in place, multiple terms representing the effects of interventions, and noise terms modelling the influence of other, unobserved, factors on *R*(*t*). The central model equation is thus:

$$\begin{array}{c} \underset{\text{Reproduction}\;\text{number}\;\text{in}\;\text{location}\;l\;\text{at}\;\text{time}\;t}{\underset{︸}{R_{l}\left(t\right)}}=\underset{R_{0}\;\text{in}\;\text{location}\;l}{\underset{︸}{R_{0,l}}}\overset{R\;\text{noise}\;\text{term}}{\overset{︷}{N_{l}\left(t\right)}}\underset{\text{Effect}\;\text{due}\;\text{to}\;\text{active}\;\text{NPIs}}{\underset{︸}{\prod_{i=1}^{I}\text{exp}(-\alpha_{i}\;x_{i,l,t})}} \end{array}$$ (1)

where *I* is the number of NPIs, *α*_*i*_ is the “effect parameter” of intervention *i*, and *x*_*i*, *l*, *t*_ are indicator variables, i.e. binary variables taking the value 1 if NPI *i* is active in location *l* at time *t*, and 0 otherwise. The reduction in *R*(*t*) associated with each NPI (the “NPI effectiveness”) can be computed as 1 − exp(−*α*_*i*_). In Brauner *et al.*, but not Sharma *et al.*, the NPI effect parameter is allowed to vary slightly between different countries (partial pooling). The noise term *N*_*l*_(*t*) varies between the models; a log-normal multiplicative factor is used in Brauner *et al.* and a random walk-based multiplicative factor in Sharma *et al.* The noise term can be intuitively thought of as a random effect that accounts for residual variation in *R*_*l*_(*t*) not captured by the NPI effects.

The time-varying reproduction number *R*_*l*_(*t*) is then used to compute the change in the number of daily new infections *D*_*l*_(*t*). This relation between reproduction number and new infections is modelled differently in the two models. The Brauner *et al.* model uses a one-to-one correspondence between *R*(*t*) and daily growth rates that holds early in a pandemic, and models separately the infections that will later become confirmed cases, and the infections that will become deaths. In contrast, the Sharma *et al.* model links *R*_*l*_(*t*) to *D*_*l*_(*t*) with a renewal process and models infections that will lead to cases/deaths jointly. The Sharma *et al.* model additionally includes additive noise on the number of infections, to account for the more fine-grained regions, and thus smaller regional infection numbers, in the data. In both models, the relation between *R*_*l*_(*t*) and *D*_*l*_(*t*) is moderated by the generation interval distribution (the generation interval is the time between successive infections in a chain of transmission).

New infections are then observed as confirmed cases or deaths, after some delay. For instance, the expected number of observed cases ${\bar{y}}_{l}^{\left(C\right)}\left(t\right)$ in location *l* at time *t* is computed as:

$$\begin{array}{c} {\bar{y}}_{l}^{\left(C\right)}\left(t\right)=\sum_{\tau =0}^{31}D_{l}\left(\tau -t\right)\cdot P_{C}\left(\text{delay}=\tau \right)\cdot \text{IAR} \end{array}$$ (2)

where *P*_*C*_ is the distribution over the delay between infection and case confirmation (truncated after 31 days because delays longer than this cutoff are very rare) and “IAR” is the infection ascertainment rate (i.e. the fraction of infected individuals that later test positive). The number of new COVID deaths is computed similarly, drawing on the delay between infection and death, and the infection fatality rate.

Finally, the observed cases $y_{l}^{\left(C\right)}\left(t\right)$ follow a negative binomial distribution with mean ${\bar{y}}_{l}^{\left(C\right)}\left(t\right)$ and an inferred dispersion parameter. This distribution reflects that small case numbers are more noisy and should therefore receive less weight. The number of observed deaths also follows a negative binomial distribution with a separate inferred dispersion parameter. Having separate dispersion parameters for cases and deaths ensures that they can be weighted differently if there is a difference in their output variance.

Both models require assumptions about priors for various variables, such as the NPI effectiveness or delay distributions. We use the same priors as in Brauner *et al.* [23] and *Sharma et al.* [24], where they have been justified (whenever possible) by reference to meta-analyses or patient line-list data.

#### 2.1.2 Estimating seasonality effects

To account for seasonality, we substitute *R*_*l*_(*t*) with $R_{l}^{\prime }\left(t\right)$ (adjusted for seasonality) and let each model infer a single seasonality amplitude parameter *γ* along with its other parameters. This minimal modification aims to preserve the demonstrated robustness of the original models [23, 24, 27]. We can thus estimate two separate seasonality effects; the final seasonality amplitude estimate is then pooled from the two models, equally sampling from their posterior distributions. Note the seasonal adjustment to *R*_*l*_(*t*) is *shared* across all locations *l* and therefore captures common dynamics between locations not explained by the location specific noise terms or NPIs.

We model seasonality as a sinusoidal multiplicative factor Γ(*t*) to *R*(*t*):

$$\begin{array}{c} \Gamma \left(t\right)=1+\gamma \;\text{sin}(2\pi \;\frac{t+d_{0}-d_{\gamma }}{365}+\frac{\pi }{2}), \end{array}$$ (3)

where *γ* is the intensity (amplitude) of the seasonal effect, *d*_*γ*_ is the day of the year of the highest seasonal effect on *R*, and *d*_0_ is the first day of the respective dataset. Note that only *γ* is a random variable, but *d*_*γ*_ is assumed fixed (see below) and *d*_0_ is a fixed property of the dataset. Our choice of the sinusoidal function for seasonality is motivated by an assumption that this function is a good fit for several of the upstream causal factors of seasonal forcing (S1 Appendix) in temperate Europe, such as temperature and humidity. The sinusoidal function, and the equivalent cosine function, are commonly employed for mathematical modelling of seasonal transmission dynamics [28–30]. In S6 Appendix, section 1.6, we examine the sensitivity of our results to using Fourier series models of varying degrees as an alternative function for seasonal forcing.

We assume a single, common seasonal effect for countries in similar climates and relative proximity along dimensions such as income, political structure, and culture. While average temperatures clearly are different between countries within the region, with a strong dependence on latitude, the amplitude of the seasonal variation is assumed to be similar. For both models, the time and location-specific *R*_*l*_(*t*) is replaced with seasonal $R_{l}^{\prime }\left(t\right)$:

$$\begin{array}{c} R_{l}^{\prime }\left(t\right)=R_{l}\left(t\right)\frac{\Gamma (t)}{\Gamma (0)}. \end{array}$$ (4)

Note that we divide Γ(*t*) by Γ(0) to have Γ(*t*)/Γ(0) = 1 at *t* = 0 and $R_{l}^{\prime }\left(0\right)=R_{l}\left(0\right)$, i.e. the seasonality multiplier is normalised to 1 at the start of the window of analysis. This implies that the priors over ${\tilde{R}}_{l}\left(0\right)$ need not be adjusted in either model. For both models, we assume an uniform prior *γ* ∼ *U* (−0.95, 0.95). (Sinusoidal seasonality is well-defined only for amplitudes −1 ≤ *γ* ≤ 1. We restrict *γ* to −0.95 ≤ *γ* ≤ 0.95 to ensure model numerical stability).

The amplitude of the cyclical seasonal variation (*γ*) can be converted to the reduction in transmission associated with going from the peak of winter to the peak of summer (i.e., peak-to-trough) as *R*(trough)/*R*(peak) = (1 − *γ*)/(1 + *γ*). Similarly, the amplitude can be directly converted to the expected reduction between adjacent seasons such as peak winter to mid-spring or mid-spring to peak summer (i.e., peak-to-mid).

Our analysis utilises January 1 as the seasonal peak day *d*_*γ*_, as this date is both close to the center of a stable range of *d*_*γ*_ in the sensitivity analysis of S6 Appendix, as well as close to January 3, the median peak date inferred by a model with variable *d*_*γ*_ in S6 Appendix. Note that while we show January 1 to be a robust choice of *d*_*γ*_, we are not aiming at determining its exact value.

#### 2.1.3 Inference

We infer the unobserved variables in our models using the No-U-Turn Sampler (NUTS) [31], a standard Markov chain Monte Carlo sampling algorithm; we use 250 tuning samples per chain. As explained in Eqs 3 and 4, we add *γ* as a fully-pooled (global) hidden variable to each of the two models and inferred in the same way as other variables. We fit each model separately, on different data (see below). For each model we obtain 5000 posterior samples in 4 independent chains (1250 in each chain). The Gelman-Rubin’s $\hat{R}$ statistic across the 4 chains was under 1.02 for all variables in both models, with no divergences.

### 2.3 Data

We fit each model separately, on the data and time period for which the model was originally created. Brauner *et al.* includes data on the implementation and lifting of several NPIs in 41 countries between 22nd January and 30th May 2020. We restrict the dataset of Brauner *et al*. to the 29 countries in the temperate European region (see S2 Appendix). Sharma *et al.* contains data on 17 NPIs in 114 subnational regions in 7 European countries (Austria, Czech Republic, England, Germany, Italy, Netherlands, Switzerland), and covers the period from 1st August 2020 to 9th January 2021. We use the Sharma *et al*. dataset without any modifications, for a total of 143 regions of analysis. We follow the same pre-processing steps as in the original datasets. In particular, for Brauner *et al*, we mask case numbers before a country had reached 100 confirmed cases and fatality numbers before a country had reached 10 deaths, to prevent bias from case/death importation (this is not necessary for the Sharma *et al.* model due to its noise on infections). For Sharma *et al*., we exclude any data points where the prevalence of variants of concern exceeded 10% during a given day, to mitigate potential bias introduced by more transmissible strains.

Data on confirmed cases and deaths were taken from the Johns Hopkins CSSE COVID-19 Dataset [32] and government websites (see Supplementary Table S4 in [24]).

## 3 Results

**Fig 2:**
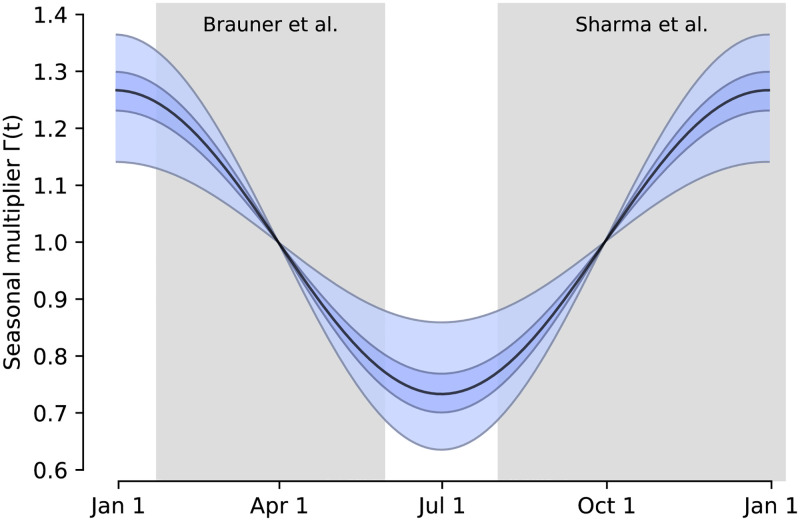
The inferred seasonal *R* multiplier Γ(*t*) of the combined models estimate, with 50% and 95% credible intervals. Gray boxes indicate data range of each dataset, i.e. 22nd January to 30th May 2020 for Brauner *et al.* and 1st August 2020 to 9th January 2021 for Sharma *et al.* The zero-width credible intervals around April 1 and October 1 owe to the fact that we model Γ(*t*) as a seasonal multiplier for *R* relative to the mid-spring and mid-fall, respectively, which implies that Γ(*t*) is assumed to be exactly equal to one for these dates.

Using two model structures and datasets on non-pharmaceutical interventions covering 72% of the 2020–2021 period in Europe, we estimate the seasonality parameter *γ* and the time-varying seasonal multiplier Γ(*t*) (Fig 2). See S3 Appendix for details.

**Fig 3:**
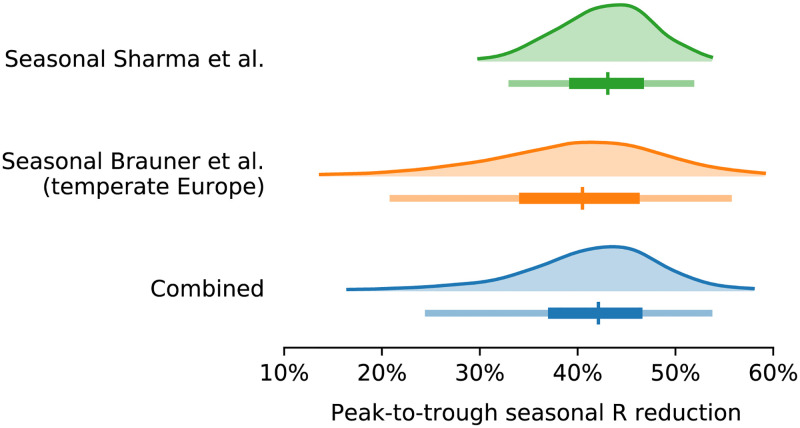
Posterior distributions of the *R* reduction on July 1 relative to January 1 with median, 50% and 95% credible intervals.

Our combined estimates from the two models are consistent with a reduction in *R* of 24.7% to 53.4% (95% CI) from January 1, the peak of winter to July 1, the peak of summer, with a median reduction of 42.1% (Fig 3 and detailed results in S3 Appendix). The combined estimate distribution is an equal-weight mixture of the posterior distributions of the two models.

**Fig 4:**
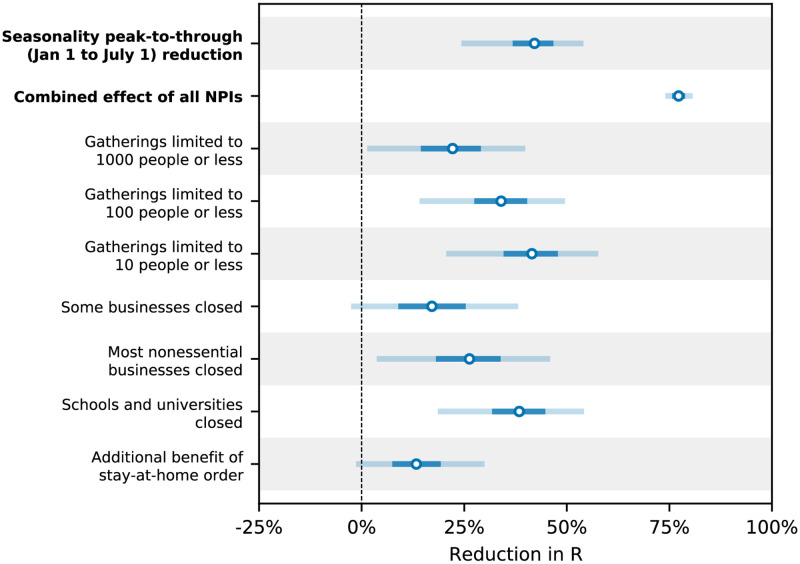
Comparison of the inferred peak-to-trough *R* reduction effect of seasonality (combined from both models) to the NPI reductions inferred by Brauner et al. [23], with 50% and 95% CIs. The seasonal effect is lower than the combined NPI effect but higher than or comparable to the individual NPI effects.

Modelling seasonality alongside non-pharmaceutical interventions allows us to gain a sense of the epidemiological importance of environmental factors. We find that the transition from winter to summer is associated with a reduction in transmission that is comparable to or greater than the effects of individual interventions, but less than the total effect of combined interventions (Fig 4).

Fig 4 compares our seasonality estimates to the original effect estimates from Brauner *et al.*, as their robustness is well-established [23, 27]. Although these estimates are based on analysis that included countries outside temperate Europe, we find that restricting our analysis to temperate regions has little effect on the inferred total effect of NPIs and thus should not invalidate the comparison (S6 Appendix, section 1.4).

Beyond NPIs, voluntary changes in behaviour and contact patterns constitute important influences on the reproduction rate. As noted, seasonal variation in behavioual patterns such as time spent indoors are an important component of our holistic conception of seasonality (S1 Fig; S1 Appendix). However, if there are behavioural changes over time that are causally unrelated with the transition between seasons, these may be mistakenly attributed to a causal effect of seasonal forcing. If, for example, voluntary personal protective behaviours beyond compliance with NPIs increased during the spring and decreased during the autumn, this would provide an alternative explanation of the respective fall and rise in transmission during those seasons. To examine whether behavioural changes over the course of the pandemic influenced our results, we incorporated country-level mobility scores into the model as an additional independent variable (see S4 Appendix). We find that our seasonality estimates are robust to adjusting for mobility trends. Specifically, our estimate of *γ* changes by less than 1.64% with the adjustment for mobility. While this finding strongly suggests that the observed seasonal pattern cannot be explained by unrelated changes in behaviour, it should be noted that data on mobility may not capture every relevant aspect of behavioural trends and that we therefore cannot conclusively rule out the possibility that our estimates are influenced by changes in behaviour.

Another important consideration is that the effect of seasonality on transmission can be mediated through many causal pathways (see S1 Fig), such as the interaction between seasonality and active interventions. For example, colder seasons may lead to increased transmission via more gatherings occurring indoors relative to outdoors. Notably, this phenomenon would be less pronounced when gatherings are banned, leading to a potential interaction between seasonality and NPI implementation. Our results provide the *average* seasonality effect across European countries in the first year of the pandemic. If countries implement very different NPIs (or none), the seasonality effect may be different as well. S6 Appendix, section 1.5, examines how our results are affected by including a term for the interaction between seasonality and NPIs; we find that neither our estimates for seasonality nor for NPIs are very sensitive to the inclusion of this interaction.

Incorporating seasonality into models of NPI effectiveness may also improve their estimates by explaining residual variation in the inferred reproduction rate. A key advance in the model proposed by Sharma *et al.* was the incorporation of a stochastic random walk process on the basic reproduction number to flexibly account for trends in transmission due to unobserved factors [24]. We find that including the seasonality term reduces the magnitude and asymmetry of the random walk considerably, thereby reducing the internal model variation (S5 Appendix). Specifically, we find that the mean square displacement (MSD) of the random walk in log-space is 0.131 for the non-seasonal model and 0.072 for the seasonal model. These results suggest a considerable amount of the residual variation can be explained by a common seasonality profile. S5 Appendix compares the NPI effects from Brauner *et al.* and Sharma *et al.* with and without the inclusion of seasonality in the model.

Estimates of seasonality and NPI effects are sensitive to modelling choices [23, 24, 27]. It is therefore vital to include a sensitivity analyses of free parameters and inputs to ensure consistent results. Relying on the demonstrated robustness of the original models, we focus primarily on the parameter that we introduce in the form of peak seasonality day. We find that the inferred mean peak-to-trough reduction in *R* varies by less than 5% across all the analysed peak seasonality dates in December and January (S6 Appendix, section 1.2). Although the seasonality magnitude is somewhat sensitive to setting the winter peak to different dates in February, these dates are considerably later in the year than the median peak date inferred in our sensitivity analysis, January 3 (see S6 Appendix, section 1.1).

Since the seasonality term we introduce is directly related to *R*_*l*_(*t*) through Eq (4), we also examine the sensitivity of our results the mean initial *R*_0_ prior. We find that our results are robust to univariate variation in this parameter, with the seasonal Sharma *et al.* model being the most sensitive (S6 Appendix, section 1.3).

## 4 Discussion

In this study, we sidestepped the intractable methodological challenge of evaluating various highly interrelated seasonal factors, and instead provide a precise estimate for the overall seasonal variation in SARS-CoV-2 transmission in temperate Europe, while adjusting for both non-pharmaceutical interventions and overall changes in mobility patterns. The strong associations we observe match the clear seasonal patterns of other respiratory viruses [2]. While reductions in reproduction rates and case numbers are not directly comparable, another recent analysis by Chen *et al.* [7] infers a 64% reduction in cases from one season to the next based on a cross-sectional regression at a single point in time, similarly suggesting a significant role of environmental factors. The general magnitude of our results is also in line with previous assumptions about the magnitude of SARS-CoV-2 seasonal forcing. For example, Kissler *et al.* assume that the reduction of SARS-CoV-2 *R*_0_ between winter and summer peaks ranges from 10% to 40% [1], while Neher *et al.* [33] assume values of *γ* between 0.3 and 0.7. Moreover, recent analyses have suggested a role of environmental factors in the B.1.1.7 lineage transmission intensity and that such factors may differentially affect the transmission of different variants of concern [6].

It is important to note that our results are not inconsistent with widespread outbreaks in warmer regions, nor do they imply that temperate regions cannot face surges in transmissions during summer periods. Despite moderate seasonal forcing, the time-varying reproduction number can remain well above 1 during the peak of the summer, particularly given high incidence of more transmissible variants such as lineage B.1.617.2 [34]. Indeed, in certain parts of Europe, *R* remained above 1 even during the warmer periods of the study window and transmission intensity currently remains high in several warmer regions across the world. Previous modelling has suggested that population immunity limits the role of environmental factors [10]. Consequently, vaccination rates, non-pharmaceutical interventions, and the prevalence of more transmissible variants will continue to be important determinants of transmission throughout the year.

Moreover, this study utilised variation in environmental and behavioural factors across time while holding the climate zone constant, and the observed results may not directly translate to comparisons across regions holding the season constant. In other words, the relationship between cooler periods and transmission within the temperate zone does not necessarily imply an exactly similar association between regional climate and transmission rates at any given point in time. This is because latitude is correlated with a wide range of epidemiological, demographic, and societal factors, each of which may affect transmission.

A major limitation of our analysis is that it relies on data from only one complete period of seasonality. We present the inferred seasonality estimates as the best estimate given the available data. Moreover, since our analysis focused exclusively on European regions in the temperate climate zone, the findings may not generalise to other climates, particularly as we have not identified the relative contributions of different causal mechanisms. Other respiratory infections show less seasonality in tropical regions relative to temperate regions as well as seasonal patterns with different peak timings, for example, during the monsoon season [2, 35]. Further research can shed light on the extent to which this is the case for SARS-CoV-2, and on the interaction between seasonality and latitude within climate regions. Moreover, further research can shed light on whether and how changes in population immunity outside our window of analysis may interact with seasonal effects.

More generally, this observational study demands caution when drawing conclusions about causality. Our analysis did not attempt to disentangle the various plausible causal pathways through which seasonality may affect transmission, and both environmental and behavioural factors can vary over the years. For example, behavioural patterns throughout the first year of the pandemic were likely exceptional, and while some behavioural changes are closely tied to modelled NPIs and thus do not bias our analysis, other relevant behavioural aspects may differ in subsequent years. Consequently, a granular focus on specific factors such as temperature, humidity, and behaviour is required for short-term prediction to inform policy.

Notwithstanding these limitations, a parsimonious form for seasonality may be adequate to understand variations over time and aid long-term policy planning. Even without disentangling the underlying factors, our approach to incorporating seasonality can augment modelling efforts to anticipate changes in transmission patterns more reliably, particularly when adjusting for important factors such as non-pharmaceutical interventions.

For such forward-looking analyses of SARS-CoV-2 seasonality, it should be noted that our inferred seasonal associations do not include two factors that play significant roles in the seasonality of other respiratory viruses. First, we treat school closures, including for holidays, as NPIs in our model due to the role of closing educational institutions in the epidemic responses of many countries. This means that any effects of closing schools are attributed to the school NPI, rather than to seasonality. This is noteworthy considering that school calendars are considered an important driver of seasonality for other respiratory viruses [19, 36]. Consequently, the full extent of seasonality would likely be greater if it is construed to include school calendars. Second, the seasonal variation of some respiratory viruses, such as influenza, owes to a combination of both the direct seasonal forcing from biological and behavioural factors as well as the indirect influence of waning population immunity [37]. Given what is known about the robustness of acquired immunity within the first year of SARS-CoV-2 infection [38], the patterns we observe likely owe almost entirely to seasonal forcing. Going forward, the long-term seasonality of SARS-CoV-2 will depend in part on developments in population immunity as well as on the emergence of variants.

Failing to account for seasonality may lead to a Panglossian outlook causing grave policy errors. For instance, a reduction in transmission over the summer could be misinterpreted as the result of population immunity alone [39], rather than the more plausible combination of immunity, seasonality, and other factors. Such an interpretation may lead to diminished support for continued vaccination efforts and booster vaccine uptake, or the premature discontinuation of non-pharmaceutical interventions. Each of these outcomes could ultimately result in an inadequate preparation for a resurgence during the colder months. At the same time, overestimating the role of environmental factors may be equally perilous. If policymakers anticipate a greater reduction in the summer months due to seasonality than will actually occur, such an interpretation could similarly lead to inadequate policy- and behavioural measures. We hope that our approach to modelling seasonality will allow for better calibrated policy responses for seasonal endemic and epidemic pathogens alike.

## Supporting information

S1 Fig Diagram of potential causal pathways for SARS-CoV-2 seasonality. A complex web of environmental, biological, and behavioural factors contribute to the seasonality of respiratory viruses. Note that this diagram excludes school calendars, as these are subsumed under non-pharmaceutical interventions for the purposes of our analysis.
(PDF)

S1 Appendix Causal pathways for SARS-COV-2 seasonality. (PDF)

S2 Appendix Datasets, model overview, and implementation. (PDF)

S3 Appendix Detailed results. (PDF)

S4 Appendix Sensitivity to adjustment for mobility. (PDF)

S5 Appendix Incorporating seasonality for NPI effect estimation. (PDF)

S6 Appendix Sensitivity analysis. (PDF)
