# Supplementary figures and images for: Seasonal variation in SARS-CoV-2 transmission in temperate climates: A Bayesian modelling study in 143 European regions
Source: PLoS Comput Biol. 2022 Aug 26;18(8):e1010435. doi: 10.1371/journal.pcbi.1010435 (PMC9455844; doi:10.1371/journal.pcbi.1010435)

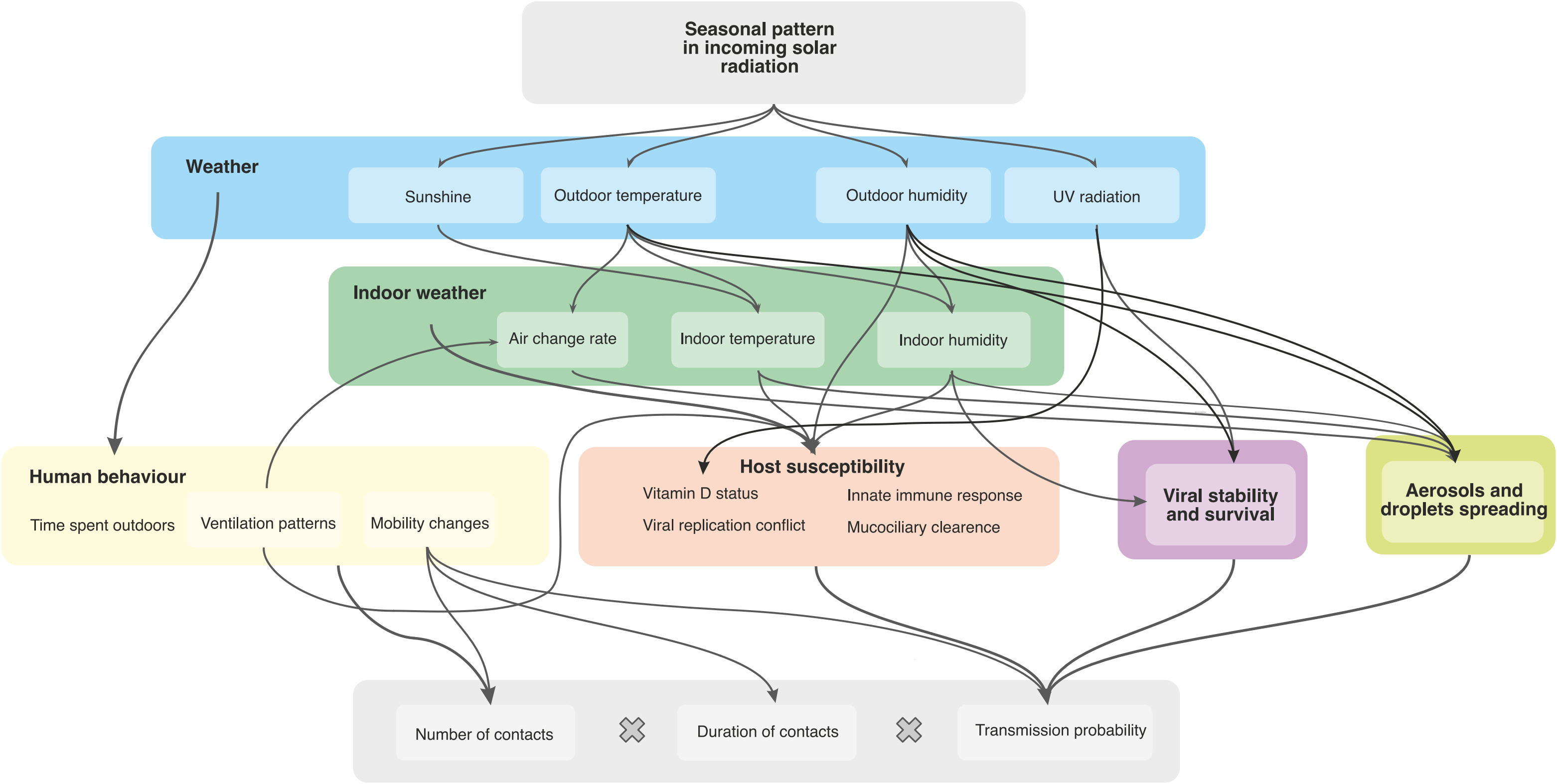

Supplement: S1 Fig — A complex web of environmental, biological, and behavioural factors contribute to the seasonality of respiratory viruses. Note that this diagram excludes school calendars, as these are subsumed under non-pharmaceutical interventions for the purposes of our analysis. (PDF) [file pcbi.1010435.s001.pdf]
