# Supplementary material for: Seasonal variation in SARS-CoV-2 transmission in temperate climates: A Bayesian modelling study in 143 European regions
Source: PLoS Comput Biol. 2022 Aug 26;18(8):e1010435. doi: 10.1371/journal.pcbi.1010435 (PMC9455844; doi:10.1371/journal.pcbi.1010435)
Supplement: S1 Appendix — (PDF) [file pcbi.1010435.s002.pdf]

1 Causal pathways for SARS-CoV-2 seasonality

1.1 Existing evidence on causal pathways

Recent reviews by Moriyama et al. [1] and Tamerius et al. [2] provide discussions of the role that each of these factors play, with a particular focus on influenza. Table 1 presents a (non-comprehensive) selection of evidence relating to some of the important causal pathways for viral seasonality. For each of these pathways, theory and evidence have been presented in support of a causal relationship. However, extensive multi-collinearities and interactions complicate any effort to tease apart the exact contributions of different factors, particularly when considering population-level transmission dynamics where experimental approaches are intractable.

As Lipsitch and Viboud succinctly put it: *"Unfortunately, this potpourri of possible mechanisms places us in a kind of Popperian purgatory, in which data in support of every hypothesis exist, yet none of the hypotheses has been subjected to tests that are rigorous enough to reject it"* [3].

**Upstream factors**	
Causal factor	Reference
Environmental factors	
Temperature	[4–8]
Absolute humidity	[4, 5, 9, 10]
Relative humidity	[11, 12]
Ultraviolet radiation	[4, 13, 14]
**Downstream factors**	
Causal factor	Reference
Host susceptibility	
Impaired innate antiviral immunity	[15, 16]
Impaired mucociliary clearance	[15, 17]
Vitamin D status	[18–20]
Viral replication conflict	[21, 21, 22]
Viral factors	
Viral survival and viability	[23, 24]
Human behaviour	
Indoor ventilation	[25, 26]
Contact rates and duration	[27, 28]
School and work calendars	[29–31]

Table 1. Selected evidence on factors driving respiratory virus seasonality.

1.2 Relating environmental parameters to the seasonal γ and collinearity between causal factors

Differences in incoming solar radiation are a common cause of seasonal changes in temperate zones, having a major effect on all other environmental parameters, such as UV radiation, temperature and humidity, which show strong correlations with the sinusoidal seasonality throughout the year.

Fig 1 shows the correlations of the seasonal Γ parameter used in the transmission model, average insolation at the top of the atmosphere, temperature, and humidity, in four of the analysed countries. The data are from the NASA POWER project [32, 33]. Country areas were selected using a rectangular bounding box minimising border overlap.

High correlation coefficients such as 0.9 indicate that including these parameters as further explanatory variables in the current analysis would lead to strong multicollinearity. Consequently, when these parameters are used as independent explanatory variables in studies of seasonal effects,

significant problems with multicollinearity are likely to create difficulties for causal inference. This poses a serious challenge for observational studies looking at individual seasonal and environmental factors and may be part of the explanation for previously inconclusive or contradictory results in the existing literature.

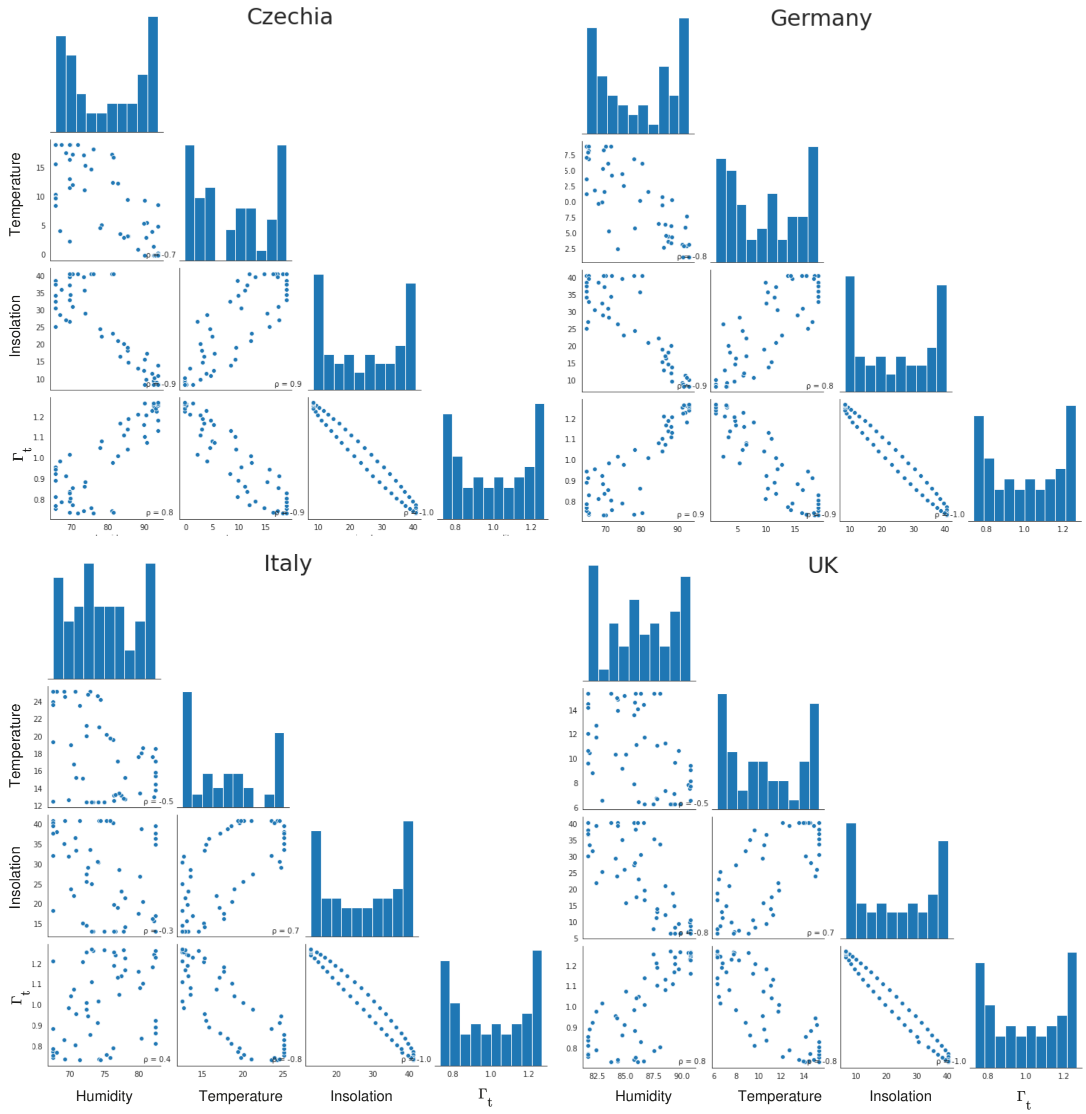

Fig 1. Correlations of the seasonal Γ parameter used in the transmission model, average insolation at the top of the atmosphere, temperature, and humidity. Each point is one weekly average value. ρ is the Pearson correlation coefficient.

It is also worth noting that the different causal pathways described in 1 depend on environmental parameters with different timescales of aggregation. For example, indoor humidity in buildings is not an instantaneous function of outdoors humidity, temperature, and ventilation, but may exhibit some inertia if walls and plaster act as a reservoir of moisture, leading to averaging effects over timescales of days, weeks, and perhaps even months. While our holistic approach focuses only on the yearly oscillation, effectively integrating over all higher frequency causal effects, studies examining correlations between, e.g., daily average humidity and daily reproduction number

are likely to miss some of the lower-frequency and delayed effects. When these longer timescale effects are included, the above described problem with multicollinearity becomes more pronounced.

References

1. Moriyama M, Hugentobler WJ, Iwasaki A. Seasonality of respiratory viral infections. Annual review of virology. 2020;7:83–101.

2. Tamerius J, Nelson MI, Zhou SZ, Viboud C, Miller MA, Alonso WJ. Global influenza seasonality: reconciling patterns across temperate and tropical regions. Environmental health perspectives. 2011;119(4):439–445.

3. Lipsitch M, Viboud C. Influenza seasonality: lifting the fog. Proceedings of the National Academy of Sciences. 2009;106(10):3645–3646.

4. Ma Y, Pei S, Shaman J, Dubrow R, Chen K. Role of meteorological factors in the transmission of SARS-CoV-2 in the United States. Nat Commun. 2021;12(3602):1–9. doi:10.1038/s41467-021-23866-7.

5. Landier J, Paireau J, Rebaudet S, Legendre E, Lehot L, Fontanet A, et al. Cold and dry winter conditions are associated with greater SARS-CoV-2 transmission at regional level in western countries during the first epidemic wave. Sci Rep. 2021;11(12756):1–13. doi:10.1038/s41598-021-91798-9.

6. Smith TP, Flaxman S, Gallinat AS, Kinosian SP, Stemkovski M, Unwin HJT, et al. Temperature and population density influence SARS-CoV-2 transmission in the absence of nonpharmaceutical interventions. Proc Natl Acad Sci USA. 2021;118(25):e2019284118. doi:10.1073/pnas.2019284118.

7. Dabisch P, Schuit M, Herzog A, Beck K, Wood S, Krause M, et al. The influence of temperature, humidity, and simulated sunlight on the infectivity of SARS-CoV-2 in aerosols. Aerosol Science and Technology. 2021;55(2):142–153.

8. Chen LD. Effects of ambient temperature and humidity on droplet lifetime–A perspective of exhalation sneeze droplets with COVID-19 virus transmission. International Journal of Hygiene and Environmental Health. 2020;229:113568.

9. Shaman J, Pitzer VE, Viboud C, Grenfell BT, Lipsitch M. Absolute humidity and the seasonal onset of influenza in the continental United States. PLoS Biol. 2010;8(2):e1000316.

10. Shaman J, Goldstein E, Lipsitch M. Absolute humidity and pandemic versus epidemic influenza. American journal of epidemiology. 2011;173(2):127–135.

11. Lowen AC, Mubareka S, Steel J, Palese P. Influenza virus transmission is dependent on relative humidity and temperature. PLoS Pathog. 2007;3(10):e151.

12. Netz RR, Eaton WA. Physics of virus transmission by speaking droplets. Proceedings of the National Academy of Sciences. 2020;117(41):25209–25211.

13. Heßling M, Hönes K, Vatter P, Lingenfelder C. Ultraviolet irradiation doses for coronavirus inactivation–review and analysis of coronavirus photoinactivation studies. GMS hygiene and infection control. 2020;15.

14. Jensen MM. Inactivation of airborne viruses by ultraviolet irradiation. Applied microbiology. 1964;12(5):418.

15. Kudo E, Song E, Yockey LJ, Rakib T, Wong PW, Homer RJ, et al. Low ambient humidity impairs barrier function and innate resistance against influenza infection. Proceedings of the National Academy of Sciences. 2019;116(22):10905–10910.

16. Foxman EF, Storer JA, Vanaja K, Levchenko A, Iwasaki A. Two interferon-independent double-stranded RNA-induced host defense strategies suppress the common cold virus at warm temperature. Proceedings of the National Academy of Sciences. 2016;113(30):8496–8501.

17. Salah B, Xuan AD, Fouilladieu J, Lockhart A, Regnard J. Nasal mucociliary transport in healthy subjects is slower when breathing dry air. European Respiratory Journal. 1988;1(9):852–855.

18. Cannell J, Vieth R, Umhau J, Holick M, Grant W, Madronich S, et al. Epidemic influenza and vitamin D. Epidemiology & Infection. 2006;134(6):1129–1140.

19. Urashima M, Segawa T, Okazaki M, Kurihara M, Wada Y, Ida H. Randomized trial of vitamin D supplementation to prevent seasonal influenza A in schoolchildren. The American journal of clinical nutrition. 2010;91(5):1255–1260.

20. Li-Ng M, Aloia J, Pollack S, Cunha B, Mikhail M, Yeh J, et al. A randomized controlled trial of vitamin D3 supplementation for the prevention of symptomatic upper respiratory tract infections. Epidemiology & Infection. 2009;137(10):1396–1404.

21. Casalegno J, Ottmann M, Bouscambert Duchamp M, Escuret V, Billaud G, Frobert E, et al. Rhinoviruses delayed the circulation of the pandemic influenza A (H1N1) 2009 virus in France. Clinical Microbiology and Infection. 2010;16(4):326–329.

22. Nickbakhsh S, Mair C, Matthews L, Reeve R, Johnson PC, Thorburn F, et al. Virus–virus interactions impact the population dynamics of influenza and the common cold. Proceedings of the National Academy of Sciences. 2019;116(52):27142–27150.

23. Shaman J, Kohn M. Absolute humidity modulates influenza survival, transmission, and seasonality. Proceedings of the National Academy of Sciences. 2009;106(9):3243–3248.

24. Polozov IV, Bezrukov L, Gawrisch K, Zimmerberg J. Progressive ordering with decreasing temperature of the phospholipids of influenza virus. Nature chemical biology. 2008;4(4):248–255.

25. Li Y, Leung GM, Tang J, Yang X, Chao C, Lin JZ, et al. Role of ventilation in airborne transmission of infectious agents in the built environment-a multidisciplinary systematic review. Indoor air. 2007;17(1):2–18.

26. Qian H, Zheng X. Ventilation control for airborne transmission of human exhaled bio-aerosols in buildings. Journal of thoracic disease. 2018;10(Suppl 19):S2295.

27. Willem L, Van Kerckhove K, Chao DL, Hens N, Beutels P. A nice day for an infection? Weather conditions and social contact patterns relevant to influenza transmission. PloS one. 2012;7(11):e48695.

28. Mossong J, Hens N, Jit M, Beutels P, Auranen K, Mikolajczyk R, et al. Social contacts and mixing patterns relevant to the spread of infectious diseases. PLoS Med. 2008;5(3):e74.

29. Ewing A, Lee EC, Viboud C, Bansal S. Contact, travel, and transmission: The impact of winter holidays on influenza dynamics in the United States. The Journal of infectious diseases. 2017;215(5):732–739.

30. Eames KT, Tilston NL, Brooks-Pollock E, Edmunds WJ. Measured dynamic social contact patterns explain the spread of H1N1v influenza. PLoS Comput Biol. 2012;8(3):e1002425.

31. Chao DL, Halloran ME, Longini IM. School opening dates predict pandemic influenza A (H1N1) outbreaks in the United States. The Journal of infectious diseases. 2010;202(6):877–880.

32. Zhang T, Chandler WS, Hoell JM, Westberg D, Whitlock CH, Stackhouse PW. A global perspective on renewable energy resources: NASA's prediction of worldwide energy resources (POWER) project. In: Proceedings of ISES World Congress 2007. Springer; 2008. p. 2636–2640.

33. POWER methodology (data parameters, sources, & validation) documentation; 2018. `https://power.larc.nasa.gov/`.
