# Supplementary material for: Seasonal variation in SARS-CoV-2 transmission in temperate climates: A Bayesian modelling study in 143 European regions
Source: PLoS Comput Biol. 2022 Aug 26;18(8):e1010435. doi: 10.1371/journal.pcbi.1010435 (PMC9455844; doi:10.1371/journal.pcbi.1010435)
Supplement: S2 Appendix — (PDF) [file pcbi.1010435.s003.pdf]

1 Datasets, model overview, and implementation

Our Brauner *et al.* seasonal model implementation is based on the Brauner *et al.* codebase [1] and can be obtained at `https://github.com/gavento/covid_seasonal_Brauner` (version tag `submitted-1`) together with the datasets used, including the temperate Europe dataset where we restrict the dataset of Brauner *et al.* [1] to the following 29 regions (out of 41 total):

Albania, Andorra, Austria, Belgium, Bosnia and Herzegovina, Bulgaria, Croatia, Czech Republic, Denmark, Estonia, France, Germany, Greece, Hungary, Ireland, Italy, Latvia, Lithuania, Malta, Netherlands, Poland, Portugal, Romania, Serbia, Slovakia, Slovenia, Spain, Switzerland, United Kingdom.

This dataset covers 22nd January to 30th May 2020.

Our Sharma *et al.* seasonal model implementation is based on the Sharma *et al.* codebase [2] and can be obtained at `https://github.com/gavento/covid_seasonal_Sharma/` (version tag `submitted-1`). We use the Sharma *et al.* dataset without any modifications and with the same preprocessing, in particular, we also exclude data points with non-negligible prevalence of novel SARS-CoV-2 variants of concern. This dataset covers the 1st August 2020 to 9th January 2021. See Figure 1 for an illustration of the covered ranges and the inferred seasonality.

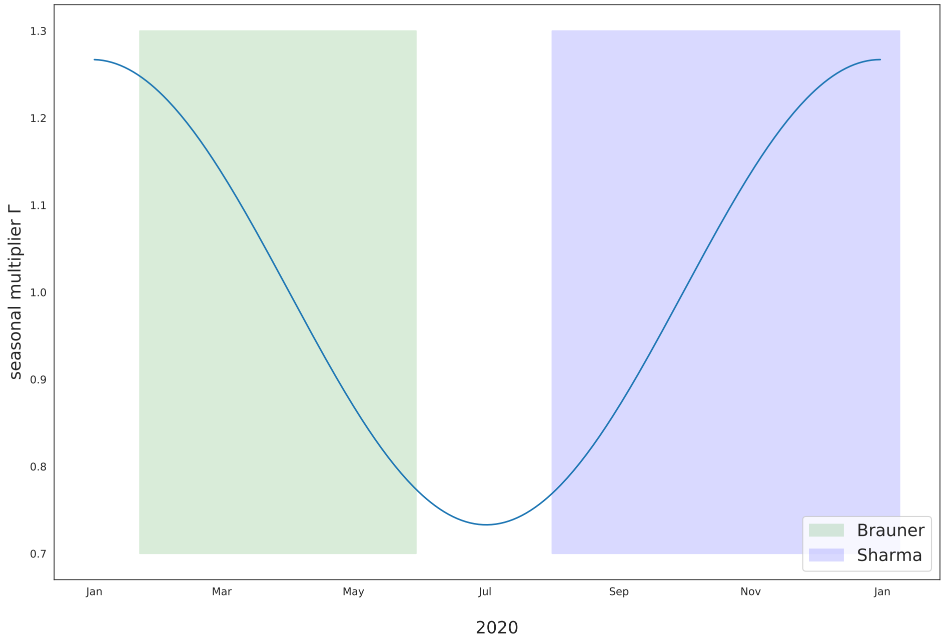

Fig 1. Study period, composed from the datasets of [1] and [2]

1.1 Model diagrams

For reader's convenience we reproduce the original model structure illustrations from Brauner *et al.* [1] and Sharma *et al.* [2]. The models are fully hierarchical partially-pooled Bayesian models, with pooling illustrated by variable grouping. We refer the interested reader to the original articles for detailed model descriptions. The seasonal models employed by us differ only by additional variable node for Γ and the resulting seasonal multiplicative term applied to $R_{t,c}$ resp. $R_{t,l}$.

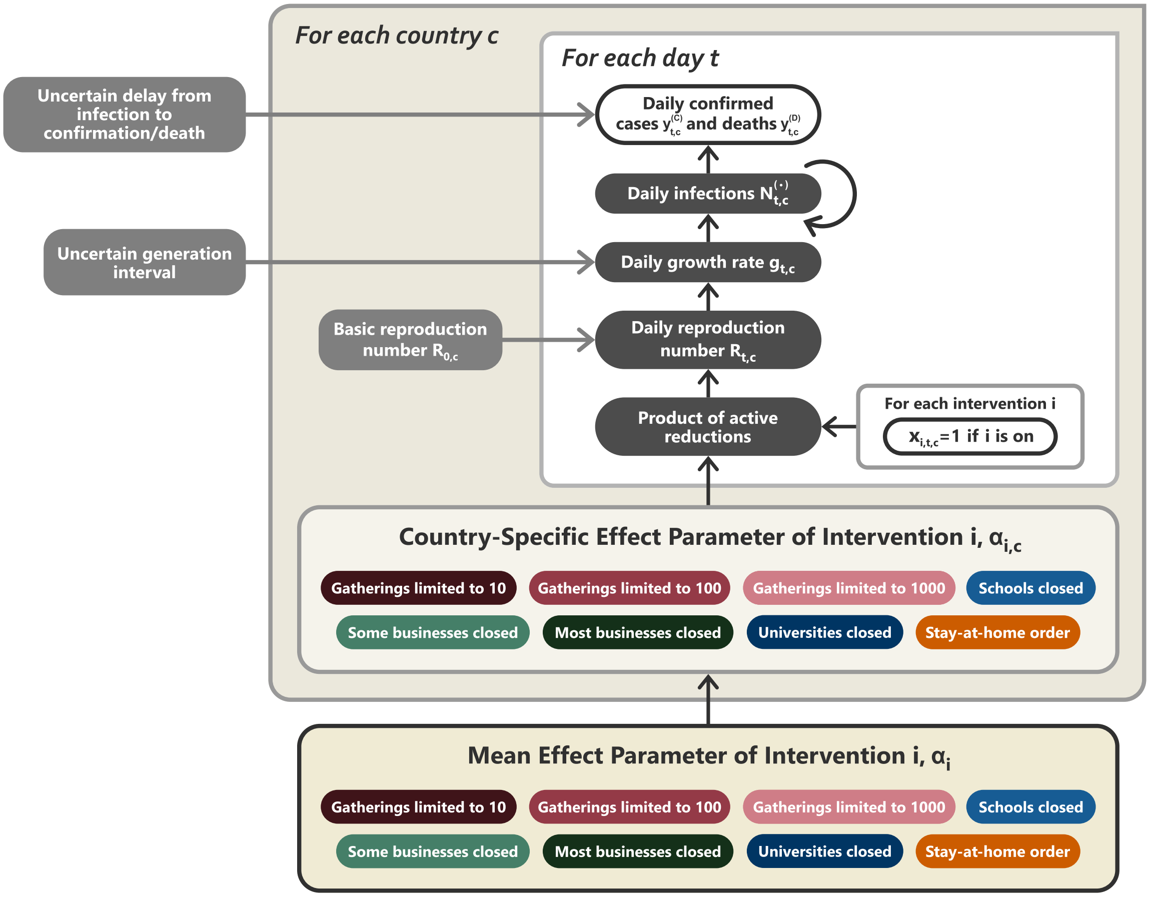

Fig 2. Model structure from Brauner *et al.* [1]. Note that c (country) plays the same role as l (location) in other model descriptions. Figure reproduced from the original paper under Creative Commons Attribution licence (CC-BY 4.0).

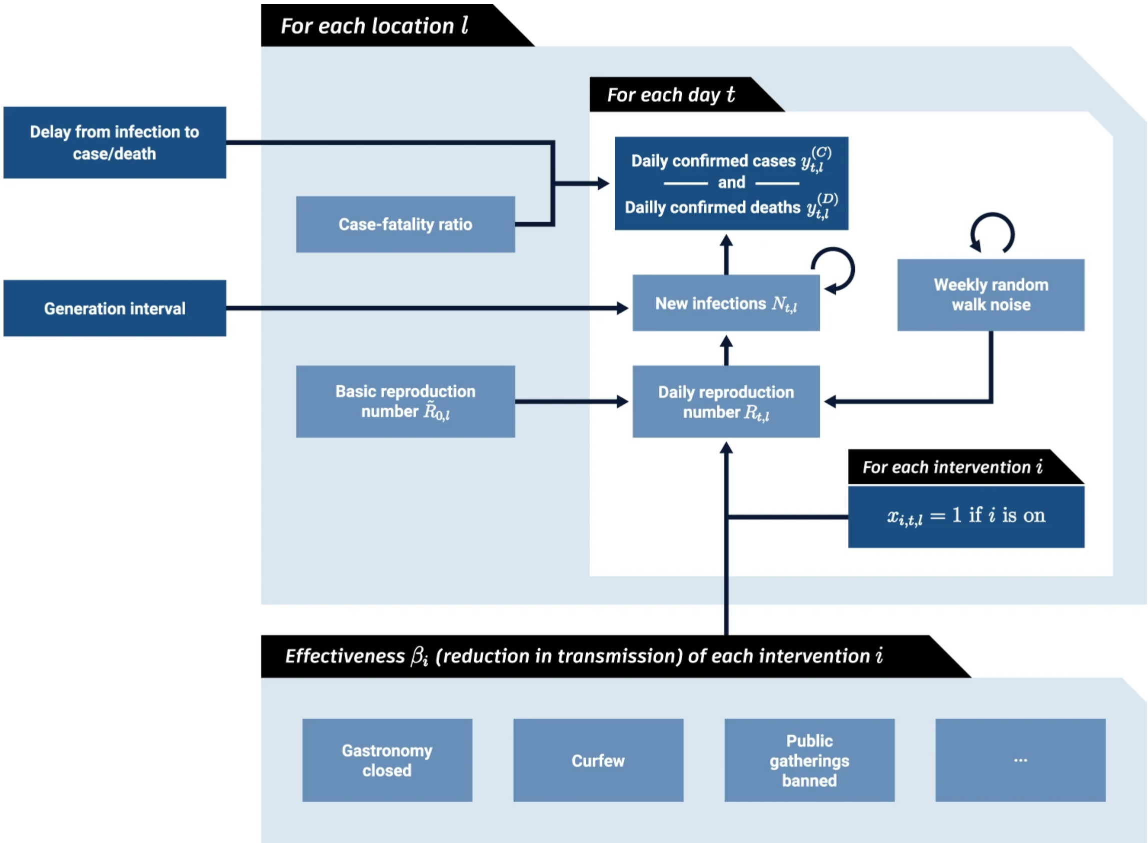

Fig 3. Model structure from Sharma *et al.* [2]. Figure reproduced from the original paper under Creative Commons Attribution licence (CC-BY 4.0).

References

1. Brauner JM, Mindermann S, Sharma M, Johnston D, Salvatier J, Gavenčiak T, et al. Inferring the effectiveness of government interventions against COVID-19. Science. 2021;371(6531). doi:10.1126/science.abd9338.

2. Sharma M, Mindermann S, Rogers-Smith C, Leech G, Snodin B, Ahuja J, et al. Understanding the effectiveness of government interventions against the resurgence of COVID-19 in Europe. Nature communications. 2021;12(1):1–13.
