# Supplementary material for: Seasonal variation in SARS-CoV-2 transmission in temperate climates: A Bayesian modelling study in 143 European regions
Source: PLoS Comput Biol. 2022 Aug 26;18(8):e1010435. doi: 10.1371/journal.pcbi.1010435 (PMC9455844; doi:10.1371/journal.pcbi.1010435)
Supplement: S3 Appendix — (PDF) [file pcbi.1010435.s004.pdf]

1 Detailed results

Model	Seasonal amplitude γ		
	**Median**	**50% CI**	**95% CI**
Seasonal Sharma *et al.*	0.275	0.248 – 0.300	0.200 – 0.348
Seasonal Brauner *et al.*	0.254	0.209 – 0.297	0.118 – 0.383
Combined	0.267	0.231 – 0.299	0.141 – 0.365

Model	Peak-to-trough R reduction [%]		
	**Median**	**50% CI**	**95% CI**
Seasonal Sharma *et al.*	43.1	39.7 – 46.2	33.3 – 51.6
Seasonal Brauner *et al.*	40.5	34.6 – 45.8	21.1 – 55.4
Combined	42.1	37.5 – 46.1	24.7 – 53.4

Table 1. Inferred median values and credible intervals of the seasonal amplitude γ and the peak-to-trough seasonality R reduction for temperate Europe countries.

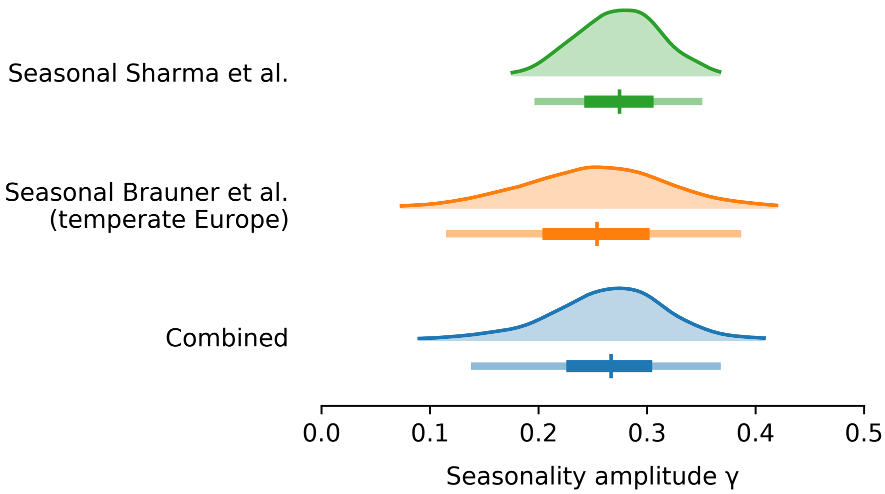

Fig 1. Posterior distributions the seasonal amplitude factor γ with 50% and 95% credible intervals.
