# Supplementary material for: Seasonal variation in SARS-CoV-2 transmission in temperate climates: A Bayesian modelling study in 143 European regions
Source: PLoS Comput Biol. 2022 Aug 26;18(8):e1010435. doi: 10.1371/journal.pcbi.1010435 (PMC9455844; doi:10.1371/journal.pcbi.1010435)
Supplement: S4 Appendix — (PDF) [file pcbi.1010435.s005.pdf]

1 Sensitivity to adjustment for mobility

Beyond NPIs and seasonality, voluntary changes in behaviour and contact patterns are important influences on the reproduction rate. As noted, seasonal variation in behavioural patterns such as time spent indoors is an important component of our holistic conception of seasonality. However, if there are behavioural changes over time that are causally unrelated with the transition between seasons, these may be mistakenly attributed to a causal effect of seasonal forcing.

To examine whether behavioural changes over the course of the pandemic biased our results, we incorporate mobility in the model by treating changes in mobility as a new distinct NPI "Mobility reduction" and run a full inference for NPIs and seasonality. We introduce the adjustment to mobility to both the Sharma *et al.* and the Brauner et *al.* models. For both models we use the country-level mobility data from Google Mobility Reports [1].

The Google Mobility Reports capture mobility change relative to a pre-pandemic, country-specific baseline in six categories: *Grocery & pharmacy, Parks, Transit stations, Retail & recreation, Residential, Workplaces.* The *Parks* category seems clearly causally related to seasonal and weather factors and we thus leave it out of the analysis.

Specifically, for the selected category set C above, each country l, and day d, we compute the activation of "Mobility Reduction" (MR) NPI as

$$x_{\mathrm{MR},d,l} = \frac{-\sum_{c\in C} M_{c,d,l}}{100|C|},$$

linearly mapping the pre-pandemic mobility level in each country to 0.0 and the (hypothetical) zero mobility to 1.0. Fig. 1 compares the posterior distributions of γ with and without adjustment for mobility.

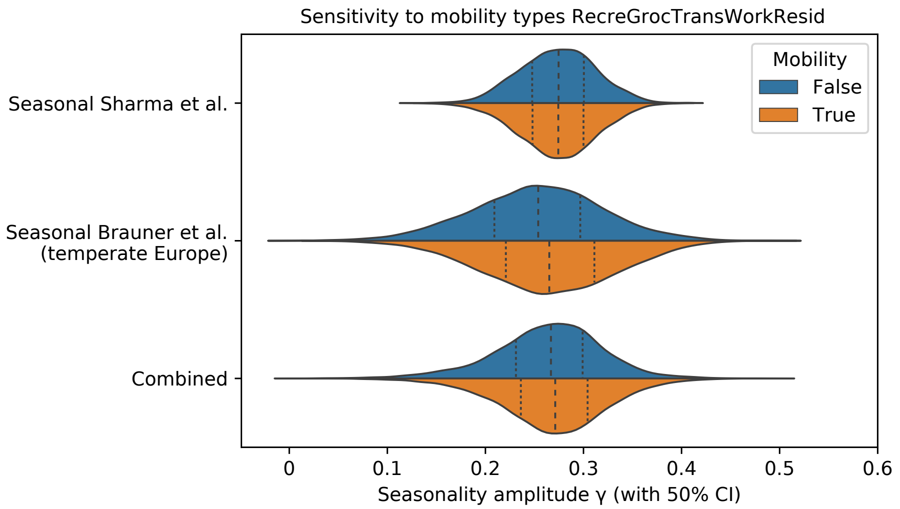

Fig 1. Comparison of posterior distributions of the seasonal amplitude γ in the main model vs with adjustment for mobility. Median and 50% credible intervals.

We find that our seasonality estimates are robust to adjusting for a combined mobility trend in locations. Specifically, our combined median estimate of γ is 0.271 with mobility vs 0.267 with the default model, a difference of 1.64%.

References

1. Google LLC. Google COVID-19 Community Mobility Reports; 2021. Available from: `https://www.google.com/covid19/mobility/`.
