# Supplementary material for: Seasonal variation in SARS-CoV-2 transmission in temperate climates: A Bayesian modelling study in 143 European regions
Source: PLoS Comput Biol. 2022 Aug 26;18(8):e1010435. doi: 10.1371/journal.pcbi.1010435 (PMC9455844; doi:10.1371/journal.pcbi.1010435)
Supplement: S5 Appendix — (PDF) [file pcbi.1010435.s006.pdf]

1 Incorporating seasonality for NPI effect estimation

1.1 Random walk noise comparison

The Sharma *et al.* model contains a random walk process on $\log N_l(t)$, the logarithm of a multiplicative factor in $R_l(t)$, in order to account for continuous slow changes of $R(t)$ through unobserved external factors such as unobserved NPIs or environmental transmission factors [1].

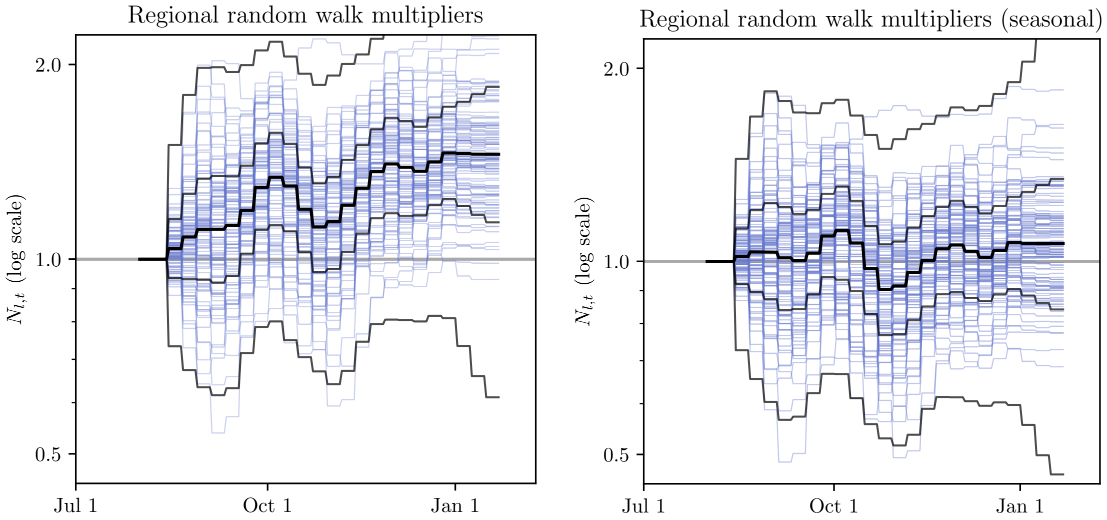

Fig 1. Inferred $N_l(t)$ for non-seasonal (left) and seasonal (right) models. Blue lines are median $N_l(t)$ for each region, black bands indicate median, 50% CI and 95% CI.

To show the effect of modelling seasonality on the inferred random walk noise, we compare $N_l(t)$ for the two models in Figure 1. While the random walk trajectories are comparable in width, the median trajectory of the non-seasonal model follows an increasing trend. Note that the random walk multiplier is modelled as a symmetrical random walk in log-space. Also note that the Sharma *et al.* model allows only weekly changes in $N_l(t)$.

To quantify the improvement, we compute the mean squared deviation (MSD) of $\log N_l(t)$, i.e. the random-walk multiplier in log-space, across the sampled random walks. We find this MSD to be 0.131 for the non-seasonal model, and 0.072 for the seasonal model, a 45% decrease. Note that here we compute MSD as

$$\mathrm{MSD}(\log N_l(t)) = \frac{1}{LNT} \sum_{l=0}^{L} \sum_{i=0}^{N} \sum_{t=0}^{T} \left(\log N_l^{(i)(t)} - \log N_l^{(i)}(t) \right)^2,$$

where $N_l^{(i)}(t)$ is the i-th sample of $N_l(t)$.

We compare $\tilde{R}_l(t)$ for the two models – the reproduction factor derived from region-specific $R_{0,l}$ by the random walk process and by seasonality effect (in the seasonal model) but *before* applying transmission reduction of the active NPIs:

$$\tilde{R}_l(t) = R_{0,l}\ N_l(t) \qquad \tilde{R}_l^{\text{seas.}}(t) = R_{0,l}^{\text{seas.}}\ N_l^{\text{seas.}}(t)\ \frac{\Gamma(t)}{\Gamma(0)},$$

where $N_l(t)$ is the random walk noise.

Figure 2 illustrates how the inferred $\tilde{R}_l(t)$ are comparable for the non-seasonal and seasonal model. However, the non-seasonal model random walk is of a larger overall amplitude and has an asymmetric trend compared to the seasonal model, as shown on Figure 1. We interpret this as an indirect evidence towards seasonality improving the quality of model fit on Sharma *et al.* data.

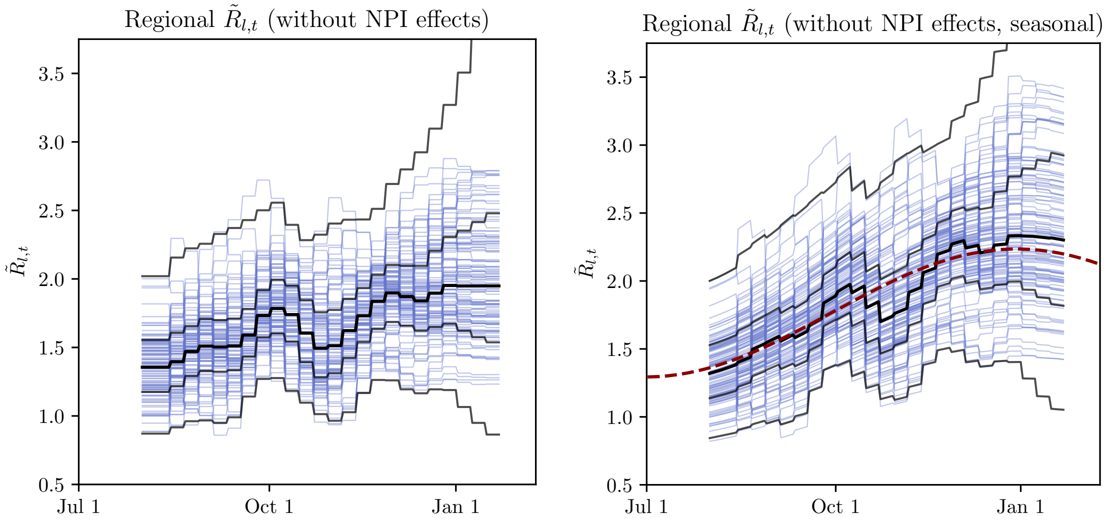

Fig 2. Inferred $\tilde{R}_l(t)$ (inferred R for the case with no interventions active) for non-seasonal *(left)* and seasonal *(right)* models. Blue lines are median $\tilde{R}_l(t)$ for each region, black bands indicate median, 50% CI and 95% CI. Red dashed line shows sinusoidal seasonality with the inferred amplitude $\gamma \approx 0.267$ applied to median of inferred $R_{0,l}$.

Note that the noise terms used in Brauner *et al.* are of different type and the model does not contain a comparable random walk noise term.

1.2 Comparison of NPI effects with and without seasonality

Figures 3 and 4 show the sensitivity of the inferred NPI effects over seasonal and non-seasonal models.

We observe that restricting the dataset regions in Brauner et al. datasets to temperate Europe ('TE', note the full dataset has regions oll over the world) has only a small influence on the inferred effects except for 'Gatherings limited to 1000 people or less', and, most notably, very little influence on the inferred combined NPI effect. The transition to the seasonal Brauner et. al. model exhibits further mild sensitivity of the 'Gatherings limited' group and robustness of the other NPI effects as well as of the combined NPI effect.

We observe that transition to the seasonal model is relatively robust and has only mild influence on the inferred NPI effects.

See S6 Appendix for further discussion of the overall NPI effect across models.

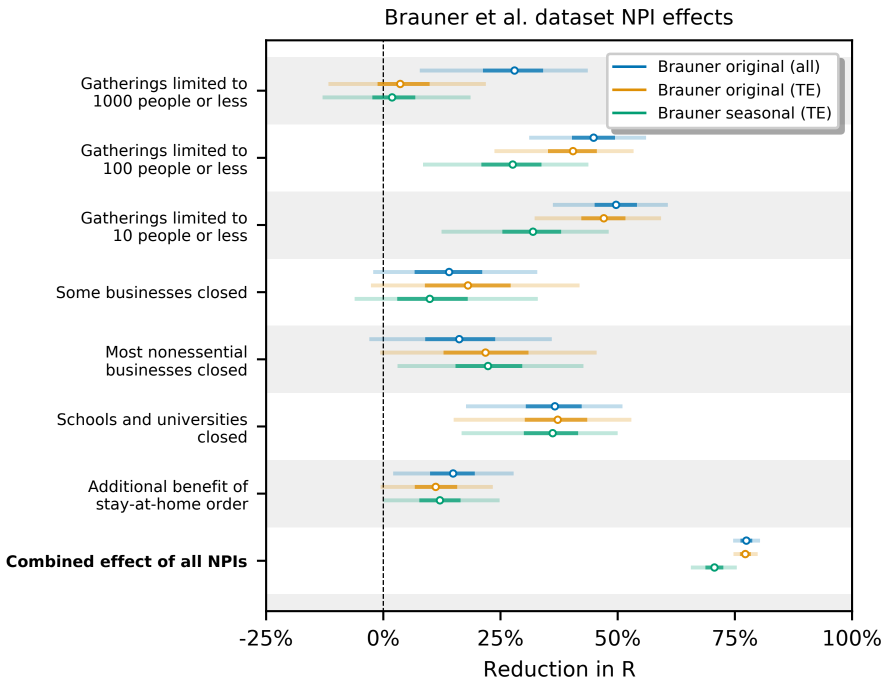

Fig 3. Comparison estimated NPI effects for the original Brauner *et al.* model –including all or just temperate (TE) countries – and its extended version including seasonality.

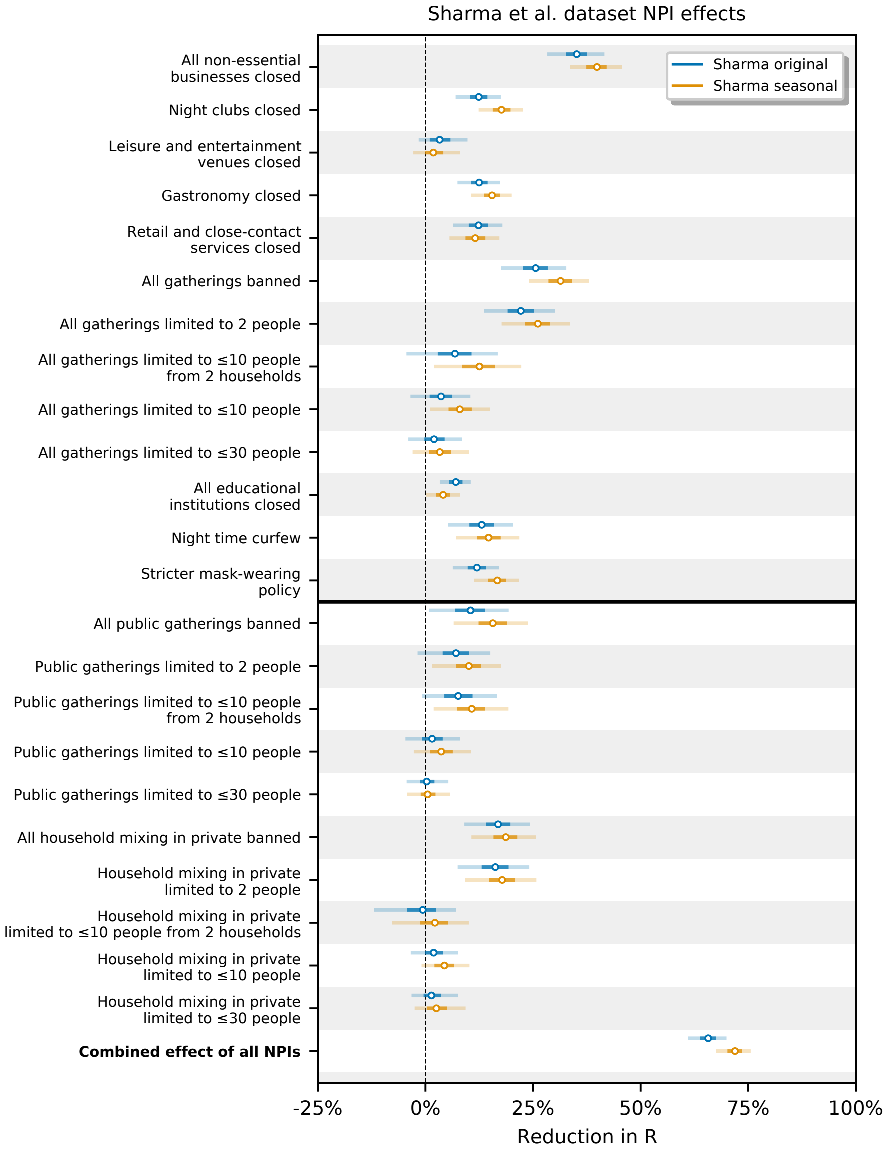

Fig 4. Comparison estimated NPI effects for the original Sharma *et al.* model and its extended version including seasonality.

References

1. Sharma M, Mindermann S, Rogers-Smith C, Leech G, Snodin B, Ahuja J, et al. Understanding the effectiveness of government interventions against the resurgence of COVID-19 in Europe. Nature communications. 2021;12(1):1–13.
