# Supplementary material for: Seasonal variation in SARS-CoV-2 transmission in temperate climates: A Bayesian modelling study in 143 European regions
Source: PLoS Comput Biol. 2022 Aug 26;18(8):e1010435. doi: 10.1371/journal.pcbi.1010435 (PMC9455844; doi:10.1371/journal.pcbi.1010435)
Supplement: S6 Appendix — (PDF) [file pcbi.1010435.s007.pdf]

1 Sensitivity analysis

1.1 Inferring the peak seasonality day

To examine the fitness of our seasonality peak estimation, we place a prior of $\mathcal{N}(\text{Jan } 1, 45^2)$ on d_γ instead of a fixed date. Figure 1 shows the distribution of the seasonality multiplier cosine curves $\Gamma(t)$ inferred with prior on d_γ. Figure 2 shows both the inferred seasonality peak day d_γ and the seasonality amplitude γ.

Note that the estimated d_γ are shown as a model validation, illustrating the range of seasonality peak the models and data are consistent with – we do not claim the models and data can infer the peak with accuracy. Note that the inferred γ in the model inferring the seasonality peak is virtually unchanged relative to a fixed seasonal peak day model (Figure 2 bottom).

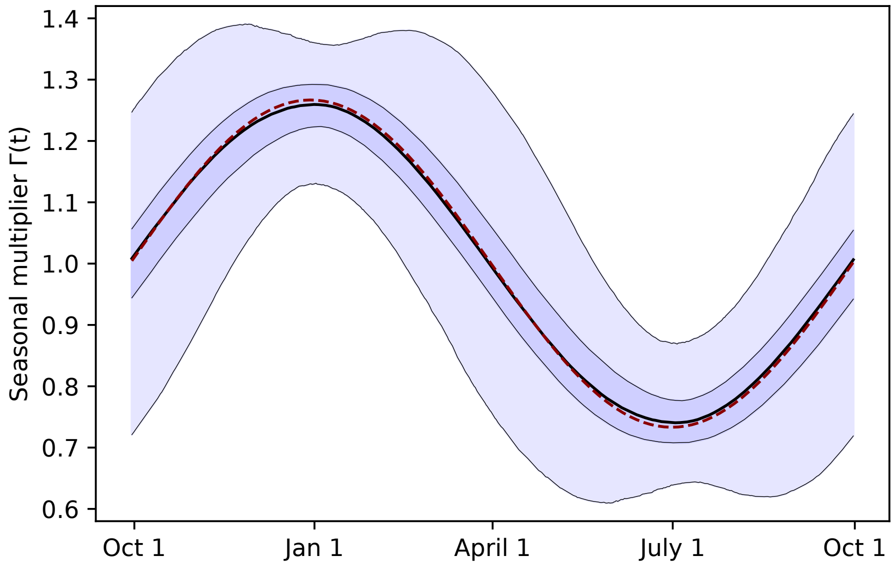

Fig 1. Distribution of $\Gamma(t)$ for the combination of Sharma et al. and Brauner et al. seasonal models with a prior on d_γ, with median and 50% and 95% CIs. The underlying $\Gamma(t)$ curves are parameterized by the joint posterior distributions on γ and d_γ. The dashed red line is the median $\Gamma(t)$ inferred with fixed $d_\gamma = \text{Jan } 1$ for comparison.

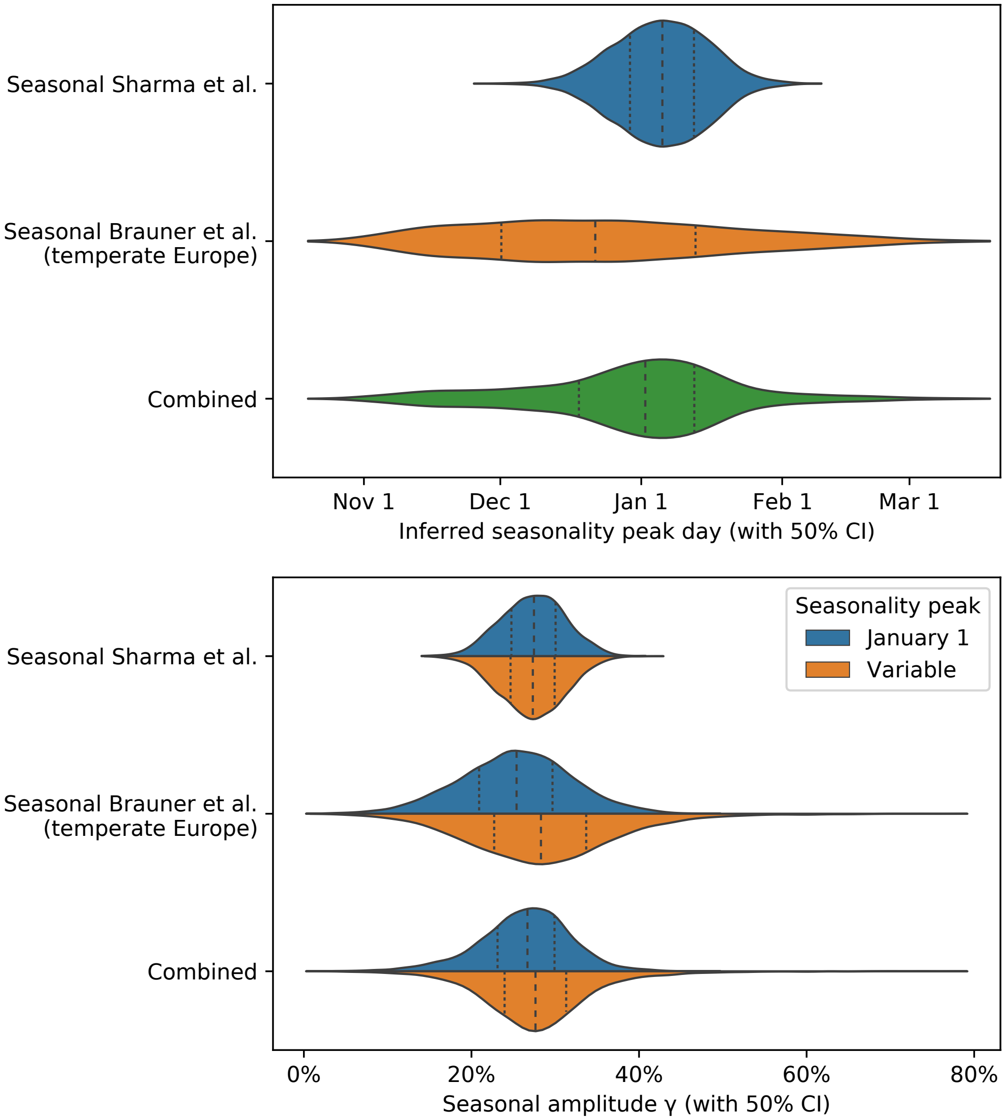

Fig 2. Inferred peak seasonality day d_γ *(top)* and γ posterior comparison in models with the peak day fixed *vs* with a variable peak day with $\mathcal{N}(\text{Jan } 1, 45^2)$ prior *(bottom)*.

1.2 Sensitivity to peak seasonality day

We test model sensitivity to the choice of peak seasonality day d_γ for $d_\gamma \in$ {Dec 4, Dec 18, Jan 1, Jan 15, Jan 29, Feb 12, Feb 26}. We observe that the inferred combined effect of the NPIs and the inferred seasonality are stable for d_γ in December and January with the exception of the combined NPI effect in Brauner et al. Note that Sharma et al. is particularly robust in this range.

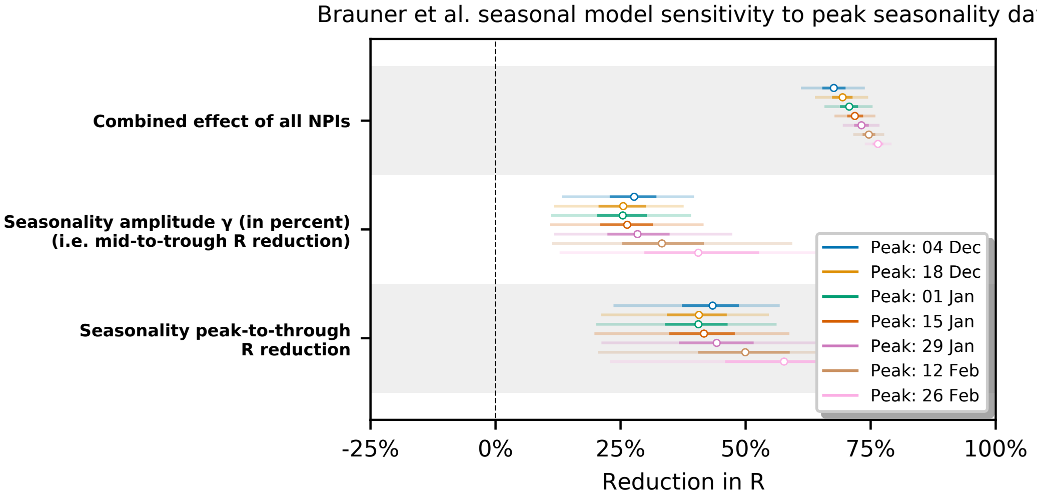

Fig 3. Sensitivity of Brauner *et al.* seasonal model to the choice of d_γ.

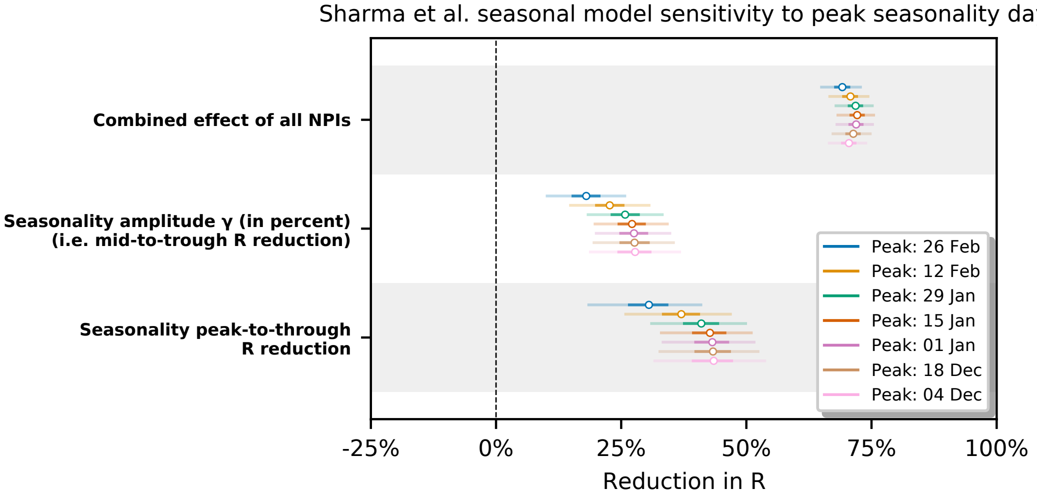

Fig 4. Sensitivity of Sharma *et al.* seasonal model to the choice of d_γ.

1.3 Sensitivity to initial R_0 prior

We test our model sensitivity to the choice of the mean of the initial $R_{0,l}$ prior (i.e. location-specific R_0 on the first day of the dataset). We analyse the $R_{0,l}$ prior mean in ranges similar to the sensitivity analyses in Sharma et al. [1] and Brauner et al. [2].

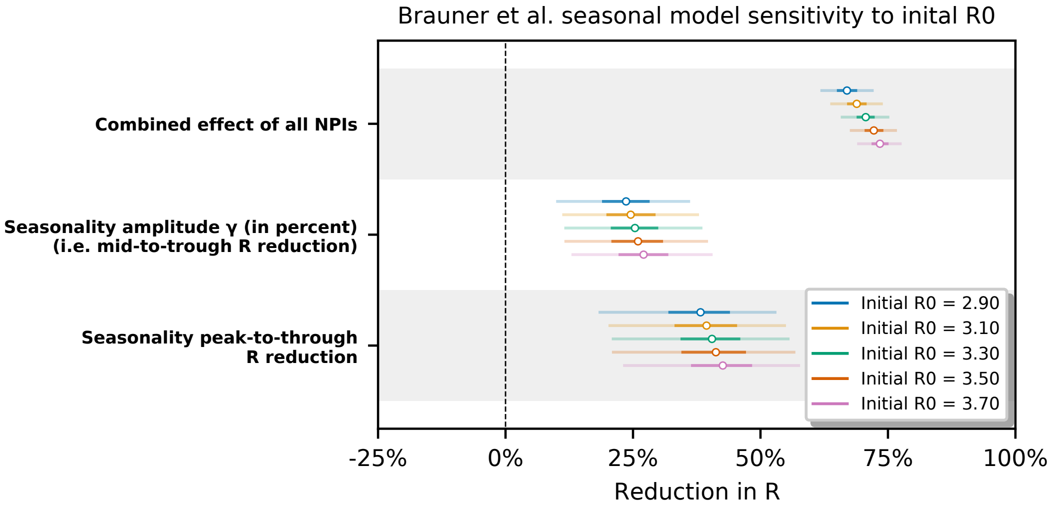

Fig 5. Sensitivity of Brauner et al. seasonal model to the initial R_0 prior mean.

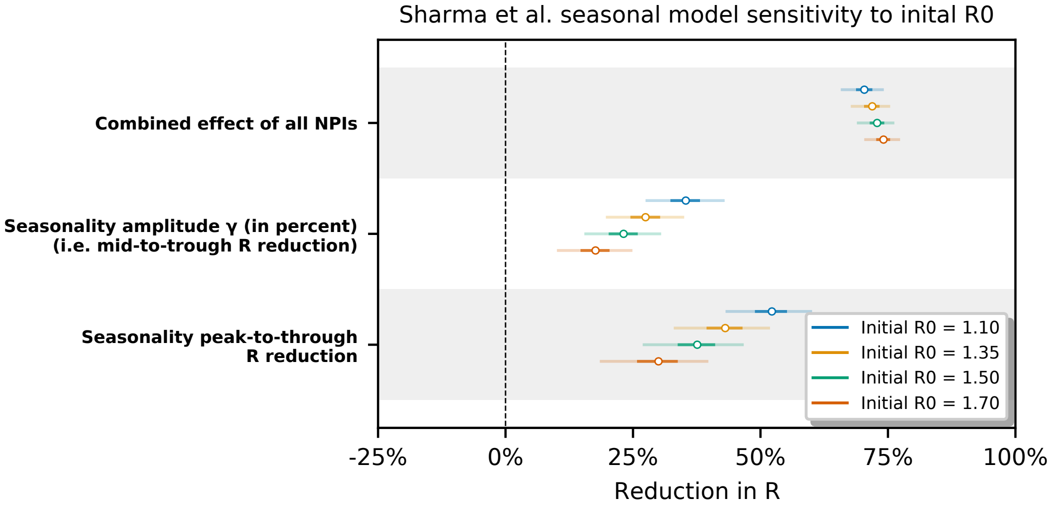

Fig 6. Sensitivity of Sharma et al. seasonal model to the initial R_0 prior mean.

This analysis is motivated by the seasonal amplitude parameter γ being closely connected with $R_{0,l}$ via Equations 1 and 4 in the main text. Mis-specifying the initial $\tilde{R}_l$ could be compensated for by the model e.g. by a different amplitude γ and therefore also the slope of $\Gamma(t)$ in the seasonality multiplier sine curve.

In Figures 5 and 6 we observe the inferred combined effect of the NPIs and the inferred seasonality to be mostly stable in the Brauner et al model. However, in the Sharma et al. seasonal model the inferred seasonality amplitude and peak-to-trough reduction are mildly sensitive to the $R_{0,l}$ prior. (Note that both those parameters are very closely tied together.)

Note that both original models also exhibit some sensitivity of the effect of NPIs to R_0 prior mean; see Fig S11 in Supplementary material of Brauner et al. [2] and Fig S13 in Supplement of Sharma et al. [1] (v1).

1.4 Inferred total NPI effects in various models

We compare the inferred total NPI effect in different models and data subsets to verify its stability: seasonal vs non-seasonal (original) models, and the original full dataset vs the dataset restricted to temperate Europe countries ("TE" in plot for Brauner et al. model).

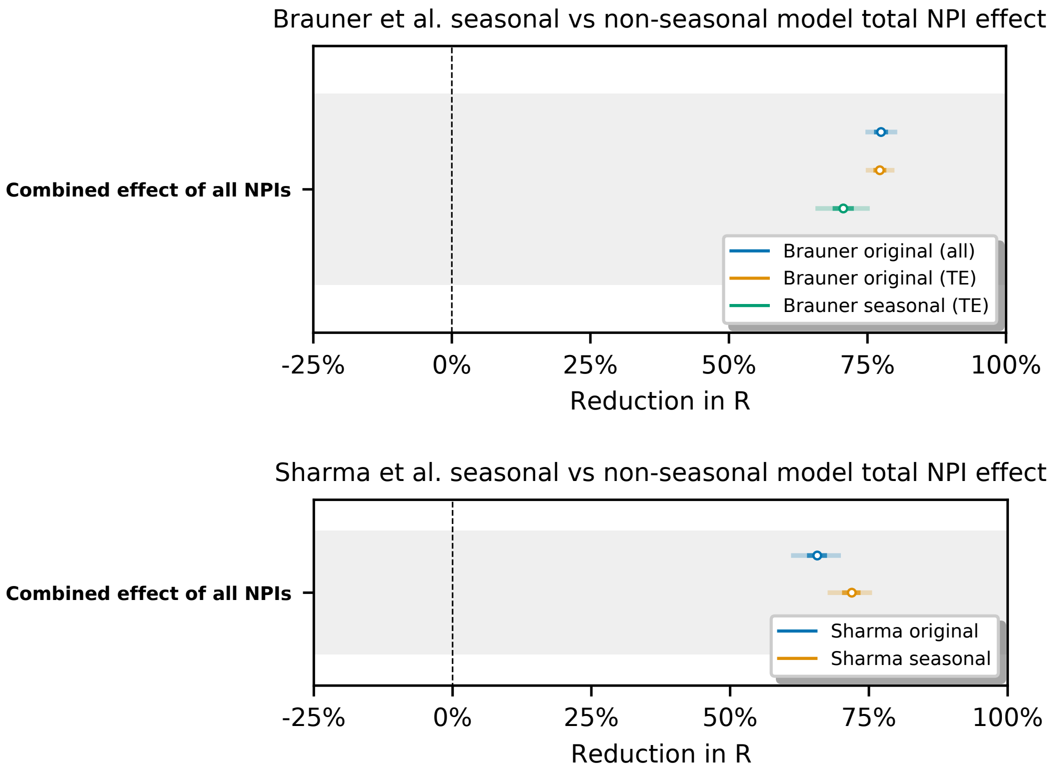

Fig 7. *Top:* Inferred NPI effects of Brauner et al. datasets with seasonal and non-seasonal (original) models. Runs marked with 'TE' are restricted to temperate Europe countries, runs marked with 'all' were run on the original Brauner et al. dataset. *Bottom:* Inferred NPI effects of Sharma et al. dataset with seasonal and non-seasonal (original) models.

Fig 7 compares the estimated NPI effects with and without seasonality for both models. Switching to a seasonal model produces a small decrease (respectively, increase) in the combined NPI effect in Brauner et al. (respectively, Sharma et al.) model. While this may be spurious, the result is consistent with a hypothesis that a part of the seasonality-related change in R_0 (i.e., the proposed spring decrease of R_0 in Brauner et al., fall increase in Sharma et al.) is in part attributed to NPI activations in both models. Recall that Brauner et al. only considers NPI activations in their model, while the Sharma et al. dataset is dominated by NPI activations compared to deactivations. However, note that both models do contain noise terms for the growth rate and other mechanisms to model small or slow changes in R due to unobserved factors, so the extent of this effect remains unclear.

1.5 Interaction of seasonality and NPI effects

One potential concern about our estimates is that they could be sensitive to including the interaction between seasonality and individual estimates of NPI-specific effects (the main inferred parameters of our model). We now examine these whether including these interactions affect our estimates of seasonality and NPI effects, using the Sharma *et al.* seasonal model and dataset. In addition to examining the robustness of our estimates, this analysis elucidates whether there are relevant interactions between seasonal forcing and specific non-pharmaceutical interventions.

Recall that the multiplicative reduction in R from NPIs is:

$$\prod_{i=1}^{I} \exp(-x_{i,l,t}\alpha_i) = \exp\left(-\sum_{i=0}^{I} x_{i,l,t}\alpha_i\right).$$

In addition to this factor, we introduce interaction terms a_i between seasonality forcing $\Gamma(t)$ and the effect α_i of active NPIs in log-space by adding the following multiplicative factor to R:

$$\exp\left(-\sum_{i=0}^{I} x_{i,l,t}\alpha_i a_i \log\Gamma(t)\right) = \prod_{\substack{i=1\\ \text{where}\\ x_{i,l,t}=1}}^{I} \exp(-\alpha_i \log\Gamma(t))^{a_i}.$$

Note that $x_{i,l,t}$ makes the log-space term zero when NPI i is not active, making the interaction model $-\alpha_i a_i \log\Gamma(t)$ conditional on NPI activation. Also note that due to the log-space representation of the expression and α_i, for consistency we need to use $\log\Gamma(t)$ rather than $\Gamma(t)$ in the interaction expression.

We introduce independent variables $a_i \sim N(0,1)$ into our Sharma *et al.* seasonal model and perform inference with otherwise standard parameters, obtaining 4 chains with $\hat{R} < 1.01$ and no divergences. The inferred seasonality amplitude is robust to the introduction of interactions with NPI effects with median $\gamma = 0.259$ (50% CI $0.24 - 0.278$), c.f. our main Sharma model which has median $\gamma = 0.275$.

Figure 8 shows the inferred seasonality forcing function in the Sharma et al. data window and for the whole year.

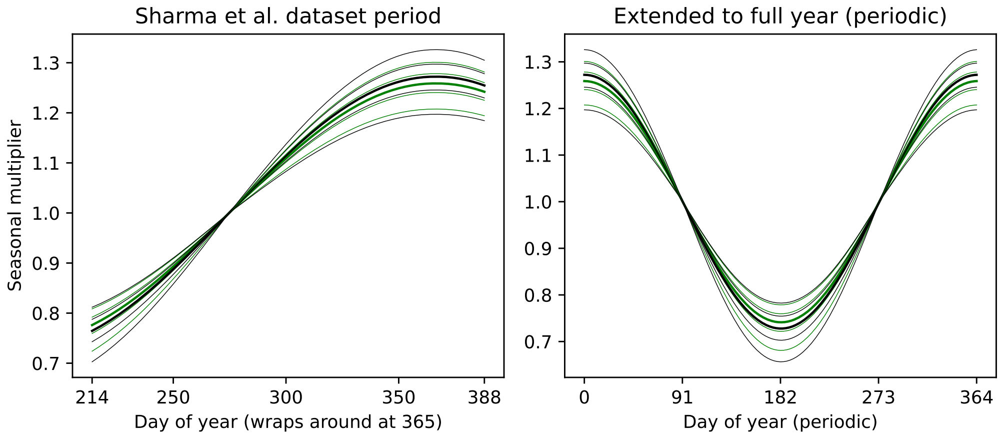

Fig 8. Inferred seasonality forcing function under interactions of NPIs with seasonality $\Gamma(t)$ (green), with seasonality forcing function without interactions (black). Each function is shown as median with 50% credible interval and 95% credible interval. The x-axis for the left-hand figure wraps around at 365, such that day 388 corresponds to day 23 of the next year.

Figure 9 summarizes the effects of the introduced interactions on the inferred values of individual NPIs. Overall, neither our seasonality estimates nor the NPI-specific estimates are very sensitive to the parameter interactions, with the exception of 'Gastronomy closure', and, to some extent, 'Night clubs closed' and 'Night time curfew'. In summary, we infer only very limited interaction between seasonality and transmission control measures.

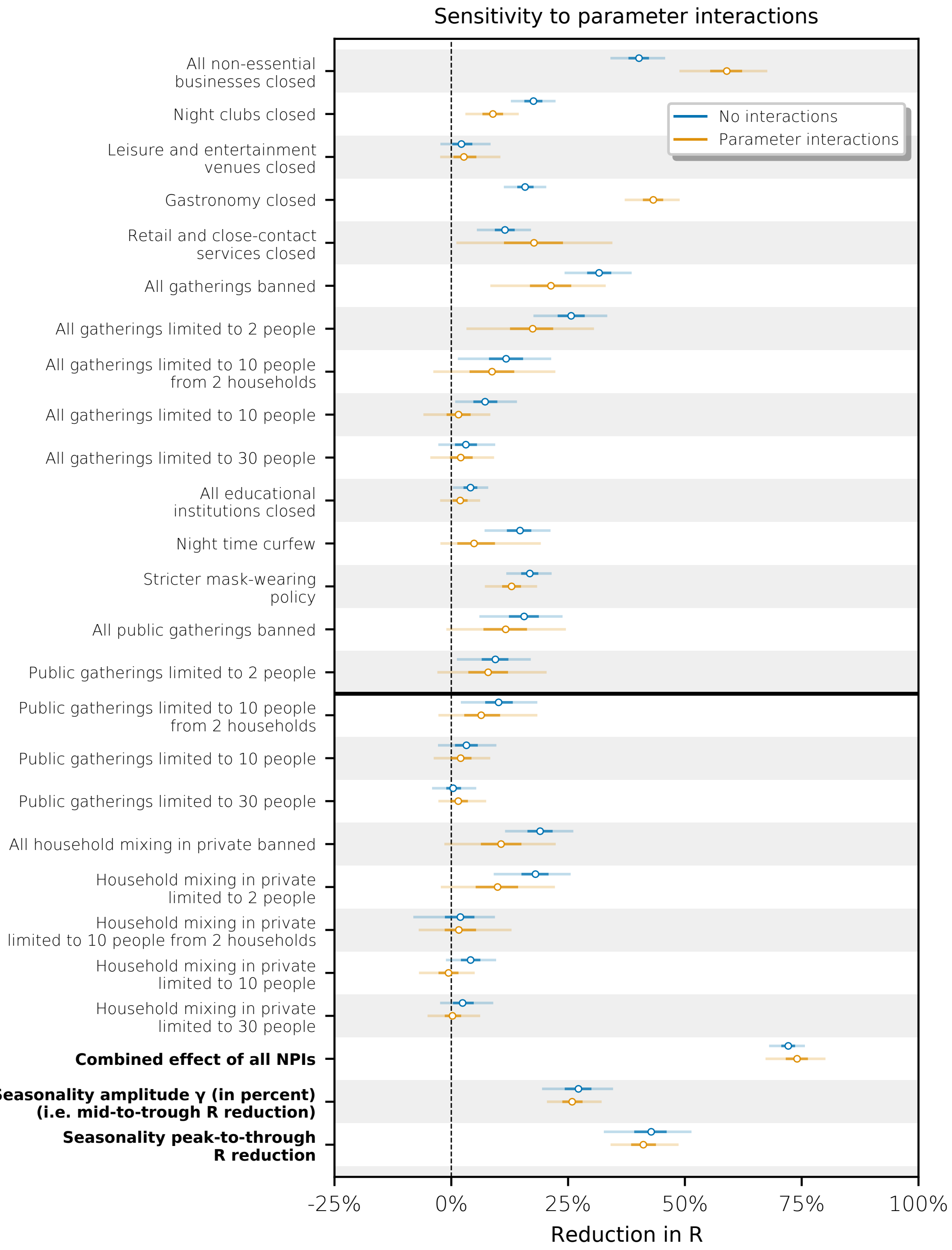

Fig 9. Sensitivity of the seasonal model based on Sharma et al. to the introduction of interactions of the seasonal forcing function with NPI effects.

1.6 Sensitivity to the choice of seasonal forcing model

To the best of our knowledge, most published work in the epidemiological literature employs a cosine function to model seasonal forcing (e.g., [3–6] all with over 200 citations). While some more complex epidemiological models use the Fourier series model of seasonality [7, 8], high-parameter approaches are susceptible to overfitting and are inadequate for time periods shorter than several years – this is also the case for modeling approaches with even more parameters, such as flexible cubic splines. Nonetheless, we analyse the sensitivity of our results to the choice of the model for seasonal forcing by considering the Fourier series model, of different degrees, as alternative models of seasonality.

The Fourier series models generalize the cosine models by adding higher cosine harmonics:

$$\Gamma_k(t) = 1 + \sum_{i=1}^{k} \gamma_i \cos(2^i \pi t + \phi_i),$$

where γ_i and ϕ_i are the amplitude and phase of the i-th harmonic, γ_1 corresponding to γ and ϕ_1 to the highest seasonality effect of our basic model.

We jointly model the phase and amplitude of the i-th Fourier term of the seasonal forcing by a complex variable $z_i \sim \mathcal{N}(0,1) + j\mathcal{N}(0,1)$ where j denotes the complex unit. With this, $\gamma_i = |z_i|$ and $\phi_i = \arg(z_i)$ corresponds to the phase of z_i. We fix ϕ_1 to correspond to the peak seasonality on January 1 as in our main analysis. Note that other $\phi_i, i > 1$ are distrubuted as the argument of a complex normal variable, that is with uniform circular distribution on $(0, 2\pi)$. We otherwise perform the analysis with the Sharma model and dataset with standard parameters; all models converge with $\hat{R} < 1.01$.

Figure 10 visualizes the difference in the inferred seasonality forcing for the analyzed period and for the full year.

Fourier series of degree 1 are virtually identical to the standard seasonality results with $\gamma = 0.272$ (50% CI $0.246 - 0.297$), compared to the estimate of $\gamma = 0.275$ for the sinusoidal Sharma model. For degrees 2 we obtain $\gamma = 0.424$ (50% CI $0.388 - 0.459$). For degree 3, $\gamma = 0.154$ (50% CI $0.106 - 0.203$). While the estimates from the degree 2 and degree 3 models differ from our sinusoidal estimates, the different estimates have overlapping and none of the credible intervals 0. For degree 3, the model seems to considerably overfit the seasonal forcing function to available data, as may be the case of degree 2.

Figure 11 summarizes the sensitivity of our NPI-specific results to the use of Fourier series as an alternative to the cosine function. In most cases, including for the combined effect of all NPIs, the inferred effects of the observed NPIs are generally not very sensitive to the choice of Fourier degree.

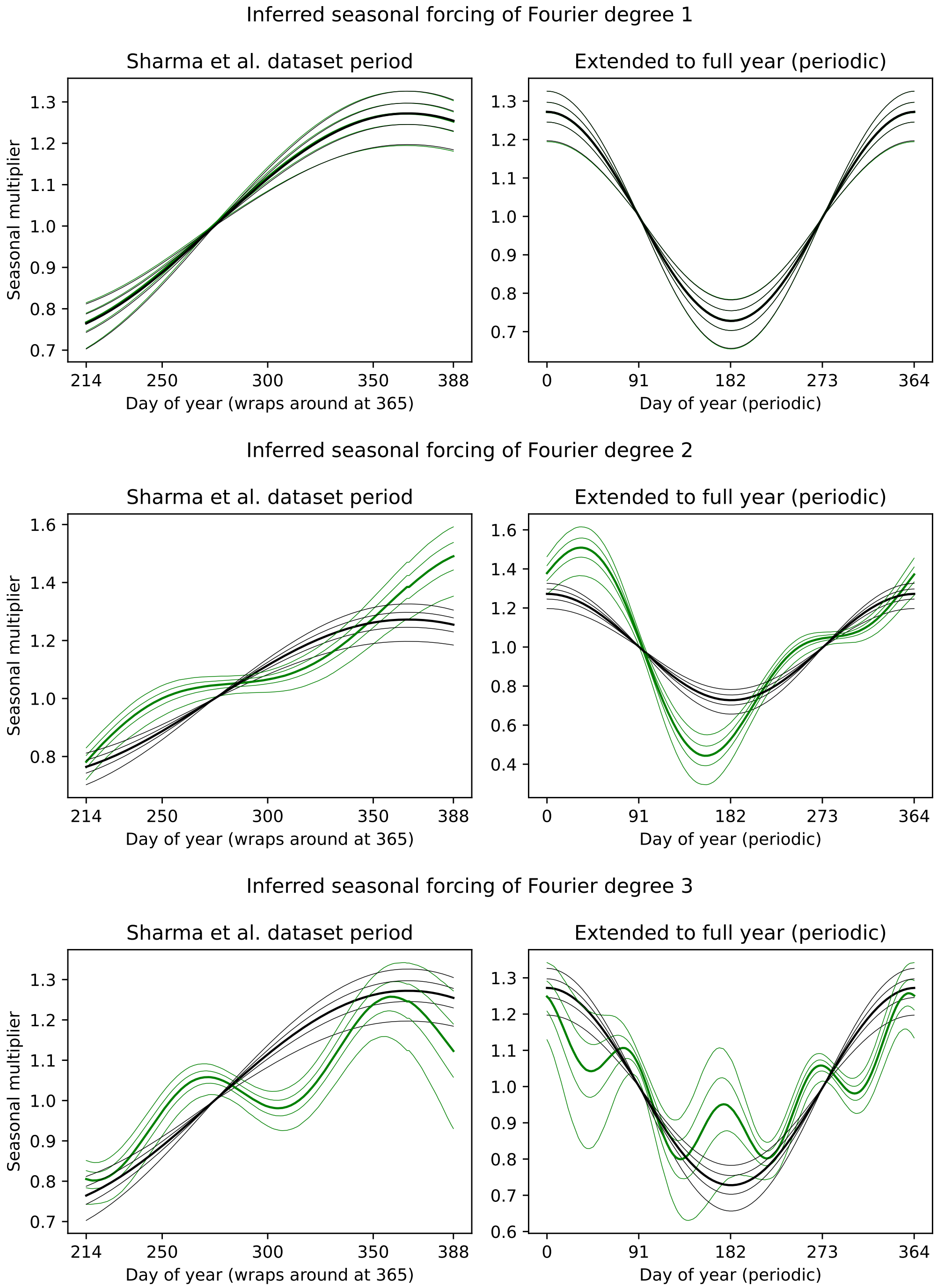

Fig 10. Green lines represent the inferred seasonal multiplier under the Fourier series model of degree 1 (top row), 2 (middle row) and 3 (bottom row), with the baseline seasonality shown in black. Each plotted with median, 50% credible intervals and 95% credible intervals. The left-hand column includes the period of the Sharma *et al.* dataset used for this analysis, while the right-hand column includes the seasonal model function plotted over the entire year. The x-axis for the right-hand column wraps around at 365, such that day 388 corresponds to day 23 of the new year.

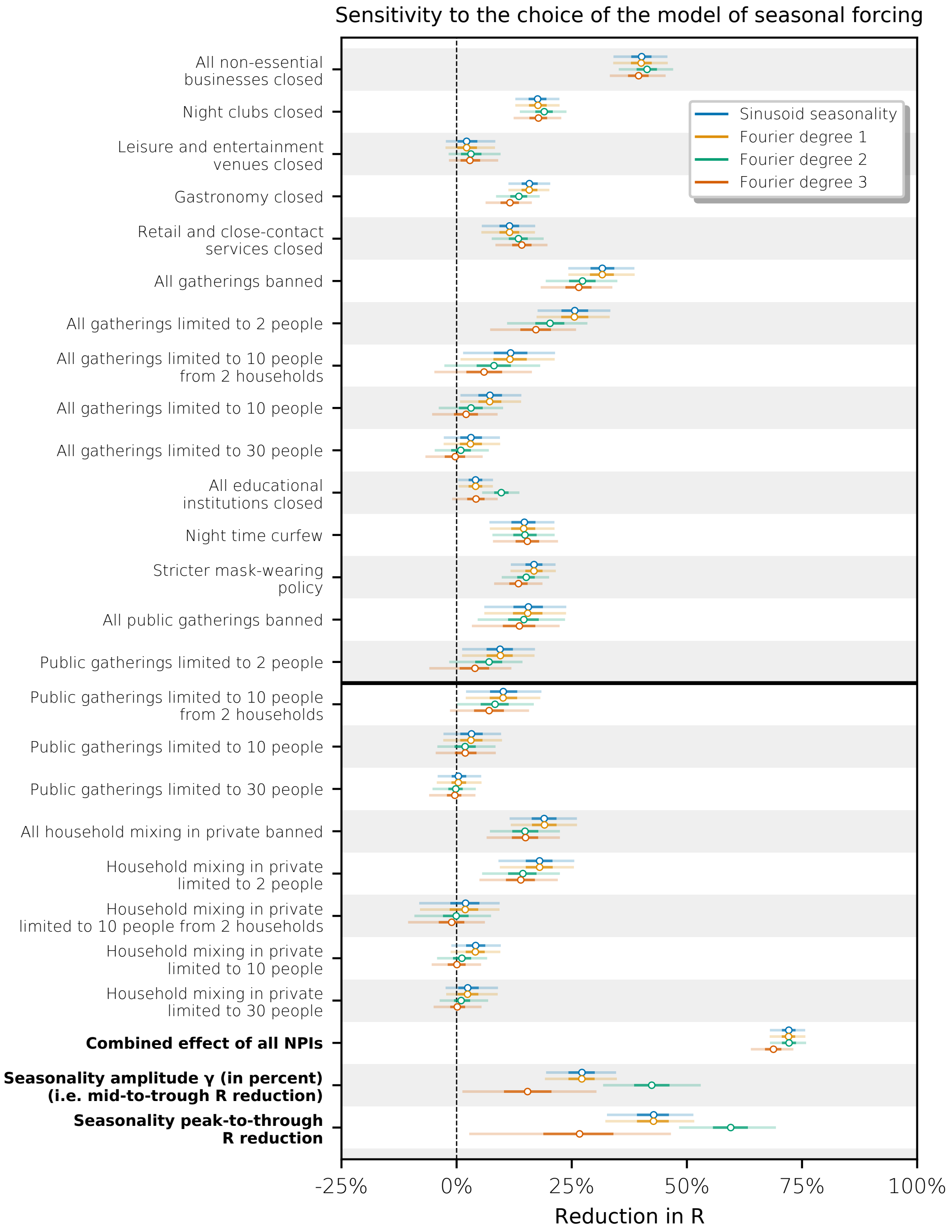

Fig 11. Sensitivity of the seasonal model based on Sharma et al. to using the Fourier series model of degrees 1, 2, and 3 as a alternative functions for seasonal forcing.

References

1. Sharma M, Mindermann S, Rogers-Smith C, Leech G, Snodin B, Ahuja J, et al. Understanding the effectiveness of government interventions against the resurgence of COVID-19 in Europe. Nature communications. 2021;12(1):1–13.

2. Brauner JM, Mindermann S, Sharma M, Johnston D, Salvatier J, Gavenčiak T, et al. Inferring the effectiveness of government interventions against COVID-19. Science. 2021;371(6531). doi:10.1126/science.abd9338.

3. Dietz K. The Incidence of Infectious Diseases under the Influence of Seasonal Fluctuations. In: Berger J, Bühler WJ, Repges R, Tautu P, editors. Mathematical Models in Medicine. Lecture Notes in Biomathematics. Berlin, Heidelberg: Springer; 1976. p. 1–15.

4. Fisman DN. Seasonality of Infectious Diseases. Annual Review of Public Health. 2007;28(1):127–143. doi:10.1146/annurev.publhealth.28.021406.144128.

5. Stone L, Olinky R, Huppert A. Seasonal Dynamics of Recurrent Epidemics. Nature. 2007;446(7135):533–536. doi:10.1038/nature05638.

6. Thompson WW, Comanor L, Shay DK. Epidemiology of Seasonal Influenza: Use of Surveillance Data and Statistical Models to Estimate the Burden of Disease. The Journal of Infectious Diseases. 2006;194(Supplement_2):S82–S91. doi:10.1086/507558.

7. Nobre FF, Monteiro ABS, Telles PR, Williamson GD. Dynamic linear model and SARIMA: a comparison of their forecasting performance in epidemiology. Statistics in Medicine. 2001;20(20):3051–3069. doi:https://doi.org/10.1002/sim.963.

8. Bhaskaran K, Gasparrini A, Hajat S, Smeeth L, Armstrong B. Time series regression studies in environmental epidemiology. International Journal of Epidemiology. 2013;42(4):1187–1195. doi:10.1093/ije/dyt092.
